# Anthocyanins, Vibrant Color Pigments, and Their Role in Skin Cancer Prevention

**DOI:** 10.3390/biomedicines8090336

**Published:** 2020-09-09

**Authors:** Zorița Diaconeasa, Ioana Știrbu, Jianbo Xiao, Nicolae Leopold, Zayde Ayvaz, Corina Danciu, Huseyin Ayvaz, Andreea Stǎnilǎ, Mǎdǎlina Nistor, Carmen Socaciu

**Affiliations:** 1 Faculty of Food Science and Technology, University of Agricultural Sciences and Veterinary Medicine, 400372 Cluj-Napoca, Romania; andreea.stanila@usamvcluj.ro (A.S.); nistor.madalina@usamvcluj.ro (M.N.); carmen.socaciu@usamvcluj.ro (C.S.); 2 Institute of Life Sciences, University of Agricultural Sciences and Veterinary Medicine, Calea Mănăştur 3-5, 400372 Cluj-Napoca, Romania; ioanaalexandrastirbu@gmail.com; 3 Faculty of Physics, Babeș-Bolyai University, Kogalniceanu 1, 400084 Cluj-Napoca, Romania; nicolae.leopold@phys.ubbcluj.ro; 4 Institute of Chinese Medical Sciences, State Key Laboratory of Quality Research in Chinese Medicine, University of Macau, Taipa, Macau 999078, China; jianboxiao@yahoo.com; 5 International Research Center for Food Nutrition and Safety, Jiangsu University, Zhenjiang 212013, China; 6 Faculty of Marine Science and Technology, Department of Marine Technology Engineering, Canakkale Onsekiz Mart University, 17100 Canakkale, Turkey; zaydealcicek@gmail.com; 7 Victor Babes University of Medicine and Pharmacy, Department of Pharmacognosy, 2 Eftimie Murgu Sq., 300041 Timisoara, Romania; corina.danciu@umft.ro; 8 Department of Food Engineering, Engineering Faculty, Canakkale Onsekiz Mart University, 17020 Canakkale, Turkey; huseyinayvaz@comu.edu.tr

**Keywords:** anthocyanins, cancer, dietary source, in vitro, melanoma, skin

## Abstract

Until today, numerous studies evaluated the topic of anthocyanins and various types of cancer, regarding the anthocyanins’ preventative and inhibitory effects, underlying molecular mechanisms, and such. However, there is no targeted review available regarding the anticarcinogenic effects of dietary anthocyanins on skin cancers. If diagnosed at the early stages, the survival rate of skin cancer is quite high. Nevertheless, the metastatic form has a short prognosis. In fact, the incidence of melanoma skin cancer, the type with high mortality, has increased exponentially over the last 30 years, causing the majority of skin cancer deaths. Malignant melanoma is considered a highly destructive type of skin cancer due to its particular capacity to grow and spread faster than any other type of cancers. Plants, in general, have been used in disease treatment for a long time, and medicinal plants are commonly a part of anticancer drugs on the market. Accordingly, this work primarily aims to emphasize the most recent improvements on the anticarcinogenic effects of anthocyanins from different plant sources, with an in-depth emphasis on melanoma skin cancer. We also briefly summarized the anthocyanin chemistry, their rich dietary sources in flowers, fruits, and vegetables, as well as their associated potential health benefits. Additionally, the importance of anthocyanins in topical applications such as their use in cosmetics is also given.

## 1. Introduction

Today, it is a generally accepted concept that including fruits and vegetables in our daily diet is beneficial for the prevention of a vast array of human diseases. Accordingly, numerous studies based on the hypothesis of the therapeutic potential of fruits and vegetables, as well as several medicinal plants, have been conducted. Following a diet that consists of fruits and vegetables has been associated with a lower frequency of diagnosed patients suffering from several pathologies such as obesity, cardiovascular and neurological diseases, diabetes, Alzheimer’s disease, and finally, yet importantly, cancer, due to their high content in phytochemicals, such as polyphenols [1,2,3]. Phytochemicals are secondary plant metabolites, known to stimulate plant reproduction as well to protect against pathogens and phytochemistry, including a systematic study of phytochemicals which has been of significant interest to researchers in the last decades [4].

Incontrovertible evidence of various health benefits arising from incorporating anthocyanins in our diet was presented by many researchers. The worldwide interest of the research community in respect to biochemical and biological aspects of anthocyanins has considerably increased not only due to their wide therapeutic potential but also due to their anticarcinogenic effects. Currently, the potential health benefits of these extraordinary molecules are strongly related to their potent antioxidant activity. Recent studies involving anthocyanin-based extracts carried out in vitro and in vivo, along with the epidemiological studies, have granted these pigments potential anticarcinogenic properties.

The most abundant compounds with antioxidant potential in the human daily diet are represented by polyphenols. There are over 15,000 polyphenols identified in nature, widely distributed not only among fruits and vegetables but also in different types of grains, oils, alcoholic and non-alcoholic beverages [5]. They may protect against an array of chronic diseases or even various cancers, as exhibited in some pre-clinical, clinical, and epidemiologic studies [1]. However, the nature and the underlying mechanisms of the most protective effects of polyphenols are still under investigation [6]. Dietary polyphenols have gained the attention of researchers worldwide owing to their antioxidant potential. They are able to protect the human organism from free-radical-induced damage to DNA and defend against the harmful UV radiation or pathogen aggressiveness. The most common polyphenols in plants that have proved potential health benefits are the well-known class of flavonoids. Furthermore, anthocyanins are natural-occurring plant pigments which are included in this class. Nearly 700 different anthocyanins have been discovered in nature [7], and they are considered the vastest and probably the most remarkable category of water-soluble pigments occurring in plants [8]. These natural plant pigments grant the color palette of flowers, leaves, fruits, and some vegetables. The red, blue, magenta, purple, and orange colors are the result of the conjugates bonds in anthocyanins structure, which are able to absorb the light at wavelengths around 500 nm [1]. The wide range of hues which an anthocyanin solution may present is not only the consequence of the pH variations. In this regard, the various structural patterns of anthocyanins play a key role as well the proportions of these diverse forms, particularly the flavylium cation under red appearance, the quinonoidal bases under violet appearance, the colorless solution or sulfite adducts, and the chalcones, which appear as yellow [9]. However, the phenomenon called “copigmentation” is the one that can offer a more elaborate explanation for the diversity of anthocyanin-derived colors observed in nature. To date, anthocyanins have had multiple applications, not only in different industrial branches, such as the textile industry, where they are used as natural colorants, but in the health sector as well, where they serve as bioactive components in the form of nutraceuticals [10].

Recently, several studies have focused on the anthocyanins’ potential, especially their anticarcinogenic potential; and numerous publications have shown the positive effects of this family of flavonoids in cancer therapy. However, to the best of our knowledge, there have been only two review papers published on the anticancer effect of anthocyanins. One of these papers overviewed the improvements on the anticancer activities of anthocyanins and the associated molecular mechanism, without targeting a specific type of cancer [11]. The other review paper was written on the preventative and inhibitory effects of anthocyanins on a specific kind of cancer: colorectal cancer [12]. On the other hand, a review paper on natural polyphenols as anticancer agents for skin cancer was recently published [1]. However, this study reviewed the polyphenols, focusing not on anthocyanins but other phenolics such as ellagitannins, quercetin, and resveratrol in purified forms (excluding total extracts and fractions). Their chemopreventive effects against skin cancer metastasis were illustrated by reviewing 34 in vivo mechanistic studies. Still, there is no comprehensive review of the anticarcinogenic effects of dietary anthocyanins on skin cancers available in the literature.

One of the leading causes of deaths among patients diagnosed with cancer is represented by skin cancer. This type of cancer is the most encountered type of cancer in the United States [13]. Depending on the cell type, skin cancers are divided into two types: cutaneous melanoma and non-melanoma. Although cutaneous melanoma, also known as malignant melanoma, is not very common compared to other skin cancers (prevalence rate of about 4%), it is the most deadly malignancy (responsible for the 80% of mortality of skin cancer), and the number of young patients diagnosed with melanoma increases faster than the number of patients diagnosed with any other types of cancer [1]. Malignant melanoma starts in melanocytes at the basal layer of the epidermis, which are the cells specialized for producing the brown pigment called melanin. Melanocytes are part of the basal cell layer of the epidermis, but they can be found in the eye as well [14]. On the other hand, non-melanoma skin cancer originates from keratinocytes of epidermis, and this cancer is also grouped into two categories: basal cell carcinoma (BCC) and squamous cell carcinoma (SCC). As opposed to the low prevalence and high mortality of malignant melanoma, BCC is the most prevalent type of skin cancer with a low mortality rate due to its low probability to metastasize [1].

One of the most critical environmental factors for melanoma development is the UV light exposure, and the risk considerably increases in lightly pigmented skin compared to darkly pigmented skin, and as people become aged [15]. To date, several in vitro studies conducted on melanoma cell lines have demonstrated that anthocyanins have the ability to induce apoptosis [16], block the cell cycle at G0/G1 phase [17], increase the oxidative stress [18], decrease the cell viability [19], cell proliferation [20] and tumor progression [21]. Moreover, the polyphenol concentrated extract from acerola was used to examine the skin-lightening effect on brownish guinea pigs, which had been subjected to controlled UVB irradiation. The results showed that an oral administration of the applied treatment effectively lightened the pigmented skin of guinea pigs. This effect might have mainly been because of the suppression of melanin biosynthesis by inhibiting the tyrosinase activity in melanocytes [22].

In this review, we aim to provide an up-to-date overview regarding anthocyanins as functional molecules and their chemopreventive effects on melanoma in vitro and in vivo as well as a comprehensive description of major sources of anthocyanins.

## 2. Anthocyanins’ Chemistry

**Figure 1:**
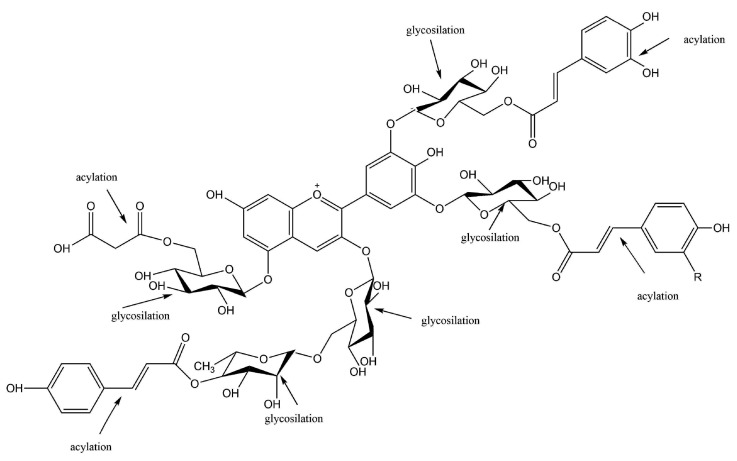
Typically glycosylation and acylation of anthocyanins (adapted from [25,26]).

Anthocyanins are water-soluble vacuolar pigments that occur ubiquitously in the plant kingdom, and they are widely distributed in fruits and vegetables as glycosides, having different sugars, such as glucose, rhamnose, xylose or arabinose, attached to an aglycon nucleus [23]. Basically, anthocyanins are glycosylated or acylglycosylated forms of polyhydroxy or polymethroxy molecules, derivates of 2-phenylbenzopyrylium cation [24] (Figure 1).

They present a characteristic chemical structure, having a carbon chain (C_6_—C_3_—C_6_) where an intermediate heterocyclic ring divides the two aromatic rings. Anthocyanin molecules may contain variations in the number of hydroxyl groups or different degrees of methylation [27]. The rich structural diversity of anthocyanins is also sustained by their nature, number, and position of sugar molecules bound to aromatic rings, as well as their nature and number of aliphatic or aromatic acids attached to sugars [27]. The sugar components of anthocyanins are usually conjugated to the anthocyanidin skeleton via the C_3_-hydroxyl group in ring C [28].

Anthocyanins are unstable molecules, and they are prone to degradation. There are several factors which may influence the color stability of anthocyanins, such as temperature, pH, enzymes activity, light, oxygen, chemical structure, anthocyanin concentration, and also the presence of complex compounds such as other flavonoids, phenolic acids, copigments, sugars and metal ions [10]. The stability of the colored structural forms of the anthocyanins and their vibrant colors is the result of copigmentation [29]. The copigmentation occurs due to molecular associations between pigments and other usually uncolored organic molecules present in the solutions that are often reported as cofactors [30]. Unlike other flavonoids, the anthocyanins carry a positive charge in acidic solution [28]. In acidic conditions, anthocyanins appear as red but turn to blue when the pH increases [10]. They all present the basic flavylium cationic structure at low pH values, and they can be distinguished from one another by the occurrence of different substituents in ring B [31].

The most common anthocyanin aglycones, also called anthocyanidins, found in plants include delphinidin (De), cyanidin (Cy), petunidin (Pt), peonidin (Pn), pelargonidin (Pg), and malvidin (Mv) [32]. Cyanidin has the highest proportion in tissues of plants (50%), followed by pelargonidin (12%), peonidin (12%), delphinidin (12%), petunidin (7%), and malvidin (7%). Nevertheless, these aglycones by themselves are very stable and convert to anthocyanins via glycosylation [10]. These structural variations among anthocyanins and anthocyanidins may influence their anticancer efficiency [20] Although a previous study demonstrated that anthocyanidins caused more potent growth inhibition of human hepatoma cell lines than anthocyanins [33], another study reported that both anthocyanins and their aglycones selectively inhibited the tumor growth [34]. Other biological effects influenced by the structural differences between anthocyanins and their aglycones are related to their antioxidant activities and bioavailability [18].

Although there has been an abundant number of studies available regarding the protective effects of dietary anthocyanins against health problems such as myocardial infarction, cardiovascular diseases, and cancer-related mortality, the exact mechanism is still complicated and not fully elucidated yet. Accordingly, a good grasp of how anthocyanins proceed in the human body from their consumption to their absorption, distribution, metabolism, and excretion is also crucial. In order to show their effect, anthocyanins need to be present in blood circulation and tissues upon their consumption through the diet. This is also known as “bioavailability,” indicating that a part of the dietary anthocyanins digested reaches the general circulation and specific locates and becomes available to exert their functions. Earlier, anthocyanins were considered to have deficient levels of bioavailability, such as between 0.26 and 1.8% in animal studies [35,36,37,38,39,40]. Nevertheless, a relevant study tracking the isotopically labeled cyanidin-3-glucoside in humans has further clarified the anthocyanin metabolism in the human body, reporting a remarkable higher recovery level of anthocyanins (12.4%) [40]. The authors stated that this unexpectedly higher bioavailability of the specific anthocyanin could be due to newly identified metabolites.

Once ingested, anthocyanins travel through the gastrointestinal tract, including stomach, small intestine, large intestine (fecal elimination), portal vein, liver, general circulation, organs, and tissues as well as being discarded through urine. As opposed to other flavonoids, previous studies have revealed that the glycosides follow a unique pattern and can be present in the human circulation after a few minutes of anthocyanin glycosides intake (reaching 100nM within 0.5–2 h and disappearing from the bloodstream in less than 6 h), indicating the intact anthocyanin absorption from the stomach [40,41,42]. Animal studies also validated the effective absorption of anthocyanins from the stomach [43,44,45]. However, the small intestine is the major part where anthocyanin absorption occurs. Various factors, including the type of aglycone, sugar moiety, or acylated groups and the presence of other flavonoids, may alter the anthocyanin absorption rate, and the extent [46,47]. Still, monitoring the intact anthocyanins may not be the best approach to evaluate the level of anthocyanin absorptions. Several studies evaluating the bioavailability of the anthocyanins have reported that along with the anthocyanins, their conjugates, and other related metabolites such as a variety of phenolic acids, might be present in the plasma [42,48]. In one human study, 35 anthocyanin metabolites were determined after labeled cyanidin-3-glucoside intake, with 17 being in the bloodstream, 31 in urine, and 28 in feces [48]. Indeed, the concentrations of phenolic acid metabolites detected in the bloodstream were remarkably higher than those of their parent anthocyanins. Thus, the health benefits of orally administered dietary anthocyanins could be due to these higher rates of metabolites [41]. On the other hand, some anthocyanins can reach the colon in substantial amounts. These anthocyanins go through decomposition there in the large intestine by the present microbiota [41]. A previous study demonstrated the hydrolyzation of the anthocyanin glycosides in intestinal microflora within 20 min to 2 h [49]. Furthermore, some studies conducted in rats and pigs in the literature also showed that anthocyanins could reach the liver, eye, and brain tissue [43,50].

## 3. Anthocyanins, as Part of the Daily Human Diet

**Figure 2:**
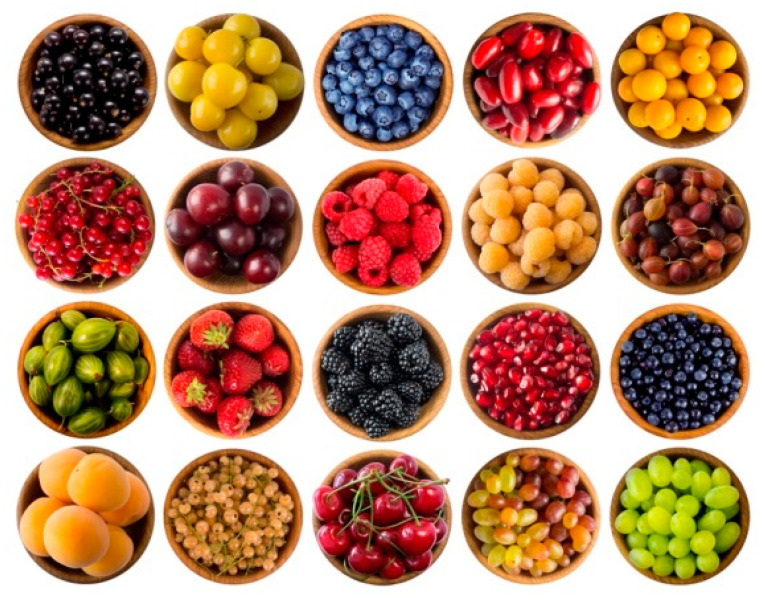
Food rich in the source of anthocyanins.

A key factor for maintaining human homeostasis is food intake, but more importantly, the nature of the food, which constitutes our diet [51]. As mentioned earlier, evidence regarding the potential health benefits provided by the consumption of fruits and vegetables was reported by many researchers. In these studies, the biggest challenge was to identify the specific compounds responsible for the health benefits. Then, the new fields of research turned their attention towards flavonoid compounds [52], mainly on anthocyanins. In the flavonoid-rich foods ingested, anthocyanins represent a large subclass [53]. Almost all berry types are abundant in anthocyanins, as well as many other dark-colored fruits and vegetables (Figure 2) [54,55,56,57], fruit-derived products such as juices, wines, and jams. Table 1, Table 2 and Table 3 summarize major flowers, berries, and vegetables as sources of anthocyanins and their quantitative occurrence, respectively.

In 2007–2008, the National Health and Nutrition Examination Survey reported the estimated dietary intake of anthocyanins to be ~11.6 ± 1.1 mg/d for individuals aged ≥20 years [1]. This number is significant compared with other flavonoids ingested, such as genistein, quercetin, and apigenin, whose daily intake was estimated at only 20–25 mg [58]. However, anthocyanin intake largely depends on dietary habits. For instance, only one serving of berries can significantly increase daily anthocyanin intake. Additionally, the daily intake of anthocyanins can be estimated from 500 mg to 1 g if flavonoid supplements are included in the diet [59].

Another important subject to discuss, considering that anthocyanins are found in a significant amount in edible foods, is their toxicity. So far, no adverse effects of anthocyanins associated with their normal dietary intake have been reported [60]. Moreover, evaluations concerning anthocyanin toxicity suggest that adverse effects may occur only if anthocyanins are ingested at extremely high levels [61]. Certainly, further complex toxicological assessments are necessary in order to have complete data regarding side-effects of different concentrations of anthocyanins. To date, there is no specific level of anthocyanin intake to be recommended for optimal health benefits; therefore, this could be a new field of research, considering the consumer demands for natural health-promoting ingredients growing to a greater extent [61].

## 4. Rich Sources of Anthocyanins

### 4.1. Flowers

**Figure 3:**
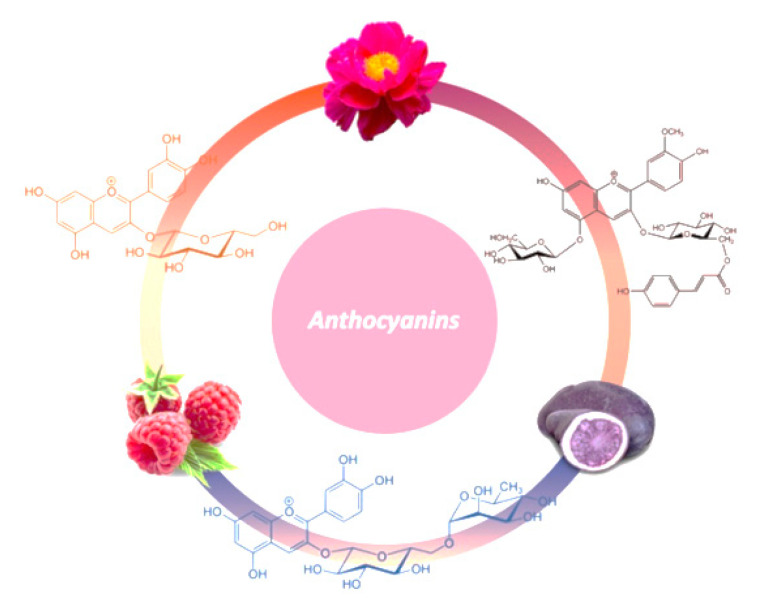
Anthocyanins’ sources and their chemical patterns.

Including edible flowers in the human diet has recently become a trend, although they have been part of human nutrition since ancient times. They are considered plant foods which proved their medicinal use during the time, as well as the various health-promoting effects, due to their content of bioactive compounds such as phenolic compounds. Various species of edible flowers are consumed as ingredients in different meals, salads or drinks. They are widely used in tea infusions as well, due to their curative properties. Reports have shown that edible flowers contain anthocyanins (Figure 3) with a wide range of functional properties, e.g., antioxidant activity. For example, species of edible flowers used as greens in salads, such as Nasturtium and Morning glory, were reported to present high anthocyanin content [62,63]. Cyanidin*-3-O-*glucoside was identified as the major anthocyanin in culinary herbs such as purple basil [64,65]. Derivates of petunidin and delphinidin were identified in saffron [66,67]. Anthocyanins were also identified in various species widely used for tea infusions, such as delphinidin derivates in *Clitoria ternatea* L. [68,69], cyanidin 3,5-di-O-glucoside in edible rose and cyanidin-3-o-sambubioside and delphinidin-3-O-sambubioside in *Hibiscus sabdariffa* [70]. Cyanidin derivates were identified in flowers as well, such as *Clemantis* [71], *Chinese herbaceous peony* [72], or *Plumeria rubra* [73] with medicinal use. In Table 1, the major anthocyanins and their concentrations in flowers are outlined.

**Table 1:** Rich sources of anthocyanins found in flowers.

Source	Major Anthocyanin Reported	Total Anthocyanins Content mg/100 g FW *	Total Anthocyanins Content mg/100 g DW **	Ref.
Purple basil *(Ocimum basilicum L.)*	Cyanidin-3-O-glucoside			[70]
Purple basil *Ocimum basilicum L. Purple Ruffles*	Cyanidin-based, p-coumaryl acid	0.0127		[64]
Butterfly peas *(Clitoria ternatea L.)*	Delphinidin derivates	NR ***	NR	[74]
Butterfly peas *(Clitoria ternatea L.)*	Delphinidin-3-O-malonyl-glucoside	NR	NR	[74]
Butterfly peas *(Clitoria ternatea L.)*	Delphinidin-3-O-(6′′-O-malonyl)-β-glucoside-3′,5′-di-O-β-glucoside	NR	NR	[68]
Camelia *(Camelia cv Dalicha)*	Cyanidin-3-O-(2-O-β-xylo- pyranosyl)-β-galactopyranoside Cyanidin-3-O-(2-O-β-xylopyranosyl-6-O-(z)-p-coumaroyl)-β-galactopyranoside	NR	NR	[75]
Camelia *(Camelia cv* Hongkongensis Japonica Hongkongensis Semiserrata Chekiangoleosa)	Cyanidin-3-O-(6-O-(e)-p-coumaroyl)-β-glucopyranoside Cyanidin-3-O-(6-O-(e)-p-coumaroyl)-β-galactopyranoside	5.6± 1.6 5.6± 2.2 48.5± 24.2 26.9± 6.0 20.5± 1.5	NR	[76]
Saffron *(Crocus sativus)*	Delphinidin-3,7-O-diglucoside		480 ± 2.33	[66]
Saffron (Crocus antalyensis)	3,7-di-O-β-d-glucoside of delphinidin Petunidin-3,7-di-O-(β-d-glucopyranoside) Delphinidin-3-O-(β-d-glucopyranoside)-5-O-(6-O-malonyl-β-d-glucopyranoside	NR	NR	[67]
Saffron (Crocus etruscus)	Delphinidin-3,5-di-O-β-glucoside Petunidin-3,5-di-O-β-glucoside			[77]
Chrysanths *(Chrysanthemum* *Dendranthema grandiflorum* *Ramat. cv Angel)*	Cyanidin-3-(3ʹʹ-malonoyl)glucoside	NR	1386± 3.9	[78]
Chrysanths *(Chrysanthemum grandiflorum* H5 Keikai Jinba)	Cyanidin-3-O-(6″-O-malonylglucoside) Cyanidin-3-O-(3″,6″-O-dimalonylglucoside)	NR	NR	[79]
Clematis *(Clematis* Niobe Madame JuliaCorrevon)	Cyanidin-3-O-β-(2′′-e-caffeoylglucopyranosyl)-(1 --> 2)-O-β-galactopyranoside-3′′-O-β-glucuronopyranoside Cyanidin-3-O-β-(2′′-e-caffeoylglucopyranosyl)-(1 --> 2)-O-β-(6′′-malonylgalactopyranoside)-3′-O-β-glucuronopyranoside Cyanidin-3-O-β-(2′-e-feruloylglucopyranosyl)-(1 --> 2)-O-β-(6′′-malonylgalactoside)-3′-O-beta-glucuronopyranoside	NR	NR	[71]
Clematis *(Clematis cv. Jackmanii Superba Fujimusume)*	Delphinidin-3-O-β-[(2”-trans-caffeoylglucopyranosyl)-(1 --> 2)-(6”-succinylgalactopyranoside)]-7-O-β-glucopyranoside Delphinidin-3-O-β-[(2”-trans-caffeoylglucopyranosyl)-(1 --> 2)-(6”-trans-caffeoyl-tartaroyl-malonylgalactopyranoside)]-7-O-β-glucopyranoside Delphinidin-3-O-β-[(2”-trans-caffeoylglucopyranosyl)-(1 --> 2)-(6”-trans-caffeoyl-tartaroyl-malonylgalactopyranoside)]-3′-O-β-glucuronopyranoside	NR	NR	[80]
Carnation *(Dianthus caryophyllus)*	3, 5-di-O-(β-glucopyranosyl) pelargonidin-6”-O-4, 6”-O-l-cyclic 3, 5-di-O-(β-glucopyranosyl) cyanidin-6”-O-4, 6”-O-l-cyclic malate	NR	NR	[81]
Carnation *(Dianthus caryophyllus cv.* Beam Cherry’ Red Vital’ Nazareno)	Pelargonidin-3,5-cyclicmalyldiglucoside Cyanidin-3-O-malylglucoside Pelargonidin-3,5-diglucoside	NR	NR	[82]
Carnation *(Dianthus caryophyllus cv.* Florigene MoondustTM F. MoonshadowTM:FMS)	Delphinidin-3,5-diglucoside-6”-O-4, 6”-O-1-cyclic-malyl diester	NR	NR	[83]
Carnation *(Dianthus caryophyllus cv.* Purple Torres statorli Maya stadofab Tasty kgr)	Cyanidin-3,5-d-O-glucosides Cyanidin-3-O-(6-O-malyl glucoside)-5-O-glucoside	NR	NR	[84]
Edible roses (An ning)	Cyanidin-3,5-di-O-glucoside	353.56 ± 2.50		[2]
Edible violet *(Viola tricolor L.)*	Delphinidin-3-(4”-p-coumaroyl)-rutinoside-5-glucoside			[85]
Freesias *(Freesia hybrida)*	Malvidin-3-O-glucoside	NR		[86]
*Roselle* *(Hibiscus sabdariffa)*	Delphinidin-3-O-sambubioside Cyanidin-3-O-sambubioside	NR	NR	[87]
	Delphinidin-3-O-sambubioside Cyanidin-3-O-sambubioside	NR		[68]
Morning glory *(Ipomoea tricolor Cav.)*	Peonidin-3-O-sophoroside-5-O-glucoside Peonidin-3-O-(2-O-(6-O-(trans-caffeoyl)-β -glucopyranosyl)-6-O-(trans-caffeoyl)-β-glucopyranoside)-5-O-(β glucopyranoside) Peonidin-3-O-(2-O-(β-glucopyranosyl)-6-O-(trans-caffeoyl)-β- glucopyranoside)-5-O-(β-glucopyranoside)	NR	NR	[68]
Leopard lily *(Iris cv.* Domestica dichotoma)	Delphinidin-3-O-(cis-p-coumaroyl)rutinoside-5-O-glucoside Delphinidin-3-O-(trans-p-coumaroyl)-rutinoside-5-O-glucoside Delphinidin-3-O-(feruloyl)rutinoside-5-O-glucoside Pelargonidin-3-O-(cis-p-coumaroyl)rutinoside-5-O-glucoside Pelargonidin-3-O-(feruloyl)rutinoside-5-O-glucoside	5.82-258.6		[88]
Japanese water iris *(Iris ensata)*	Malvidin-3-O-(p coumaroyl)rhamnosylglucoside-5-O-glucosides Petunidin-3-O-(p-coumaroyl)rhamnosylglucoside-5-O-glucosides	NR	NR	[89]
Dutch iris *(Iris hollandica)*	Delphinidin-3-O-(p-coumaroyl)rhammnosylglucoside-5-O-glucoside	NR	NR	[90]
Crimean iris *(Iris lutescens)*	Delphinidin-3-O-(p-coumaroylrutinoside)-5-O-glucoside	NR	NR	[1]
Edging lobelia *(Lobelia erinus* *cv Rosamond)*	Cyanidin-3-O-(6-O-(4-O-trans-p-coumaryl-α-l-rhamnopyranosyl)-β-d-glucopyranoside)-5- O-(6-O-malonyl-β-d glucopyranoside)-3′-O-(6-O-trans-caffeyl-β-d-glucopyranoside) Cyanidin-3-O-rutinoside-5,3′-diglucoside	NR	NR	[91]
Edging lobelia *(Lobelia erinus cv.* Aqua Blue Aqua Lavender)	Delphinidin-3-O-p-coumaroylrutinoside-5-O-malonylglucoside-3′5′-O-dihydroxycinnamoylglucoside Delphinidin-3-O-glucoside	NR	NR	[26]
Meadow/bloody crane’s-bill *(Geranium* Sanguimeum Johnson’s blue Pretense)	Malvidin-3-O-β-d-glucopyranoside-5-O-β-d-[6-O-acetylglucopyranoside]	NR	NR	[92]
Peony *(Paeonia* lactifora mlokosewitschii tenuifolian)	Peonidin-3,5-di-O-glucoside Cyanidin-3-O-glucosid	NR	NR	[93]
Peony Paeonia suffruticosa cv.Gunpohden’ Paeonia tenuifolia	Peonidin-3,5-di-O-glucoside Cyanidin-3-O-glucoside	NR	NR	[94]
Petunia *(Petunia hybrida* AN1 (R27) an1 (W225))	Cyanidin-3-O-glucoside	7.72± 0.8 µmolgFW 5.61± 0.39	NR	[95]
Petunia *(Petunia exserta)*	Cyanidin-3-O-sophoroside Cyanidin-3-O-glucoside Peonidin-3-O-glucoside	NR	NR	[96]
	Cyanidin-3-O-glucoside	NR	NR	[97]
Petunia *(Petunia hybrida)*	Peonidin-3-O-(6-(6-coumaryl rhamnosyl)-glucoside)-5-O-glucoside	NR	NR	[98]
Red frangipani *(Plumeria rubra)*	Cyanidin-3-O-β-(2”-glucopyranosyl-O-β-galactopyranoside)	NR	NR	[73]
Pomegranate *(Punica granatum)*	Pelargonidin-3-O-glucoide Pelargonidin-3-O-diglucoside	NR	NR	[99]
Korean edible rose *(Rosa hybrida cv. Noblered)*	Cyanidin-3,5-di-O-glucoside	375+_9.6	NR	[100]
*Rosa rugosa* *Hunchun* *Jiaomeisanbian* *Miaoyu*	Peonidin-3,5-di-O-glucoside Cyanidin-3,5-di-O-glucoside Cyanidin-3,5-di-O-glucoside	165 ± 17.2 44.95 ± 0.38 14.01 ± 0.61	NR	[101]
Nasturtium *(Tropaeolum majus)*	Delphinidin-3-O-dihexoside Pelargonidin-3-O-sophoroside	245.5±167.3 880.3± 18.3	31.9± 21.7 114.5±17.22	[62]
Nasturtium *(Tropaeolum majus)*	Pelargonidin-3-O-sophoroside	NR	NR	[102]
Marigold *(Tagetes erecta)*	Cyanidin-di-hexoside	NR	NR	
Aracress *(Spilanthes oleracea)*	Cyanidin-3-O-glucoside Delphinidina-3-O-glucuronide	NR	NR	
Tulip *(Tulipa fosteriana ‘Albert heijn’)*	Pelargonidin-3-O-acetylrutinoside Cyanidin-3-O-rutinoside	NR	NR	[103]
Blue periwinkle *(Vinca. major L.)*	Delphinidin-3-O-[6-O-(α-rhamnopyranosyl)-β-galactopyranoside]-7-O-(α-rhamnopyranoside)	NR	NR	[104]
Blue periwinkle *(Vinca. minor L.)*	Delphinidin-3-O-[2-O- (β-xylopyranosyl)-6-O-(α-rhamnopyranosyl)-β-galactopyranoside]-7-O-(α-rhamnopyranoside)	NR	NR	
Garden pansy *(Viola wittrockiana)*	Delphinidin-3-O-rhamnosyl-glucoside	NR	0.57 ± 1.2	[105]
Yunnan edible rose (*An ning*)	Cyanidin-3,5-O-diglucoside Cyanidin-3-O-glucoside	353.56 ± 2.50	NR	[106]
False shamrock *(Oxalis triangularis)*	Malvidin-3-O-(6-O-(4-O-malonyl-a-rhamnopyranosyl)-β-glucopyranoside)-5-O-β-glucopyranoside Malvidin-3-O-(6-O-(4-O-malonyl-a-rhamnopyranosyl)-β-glucopyranoside)-5-O-β-glucopyranoside	NR	NR	[107]
	Malvidin-3-O-rutinoside-5-O-glucoside.	NR	NR	[108]
	Mavidin-3-O-(6-O-(4-O-malonyl-a-rhamnopyranosyl-β-glucopyranoside)-5-O-b-glucopyranoside	NR		[109]

* FW—fresh weight; ** DW—dry weight; *** NR—not recorded.

### 4.2. Fruits and Vegetables

The current interest in dietary polyphenols is sustained by the mounting evidence that the intake of these phytochemicals has various human health benefits. Their uptake can be increased by consuming plant-based foods, which represent the primary source of polyphenols; however, these sources vary with the habitual diet. For example, polyphenols are major dietary components for people who drink red wine on a routine basis. The complexity of polyphenols lies in the potential for multiple decorations with other groups, including sugars, alcohols, and acids [110]. The most common flavonoids, a major sub-group of polyphenols, are rarely found in food without attached sugars, acids, or alcohols. Anthocyanidins, for instance, are mostly found as anthocyanins [111,112]. Anthocyanins represent a variety that has enhanced levels of specific polyphenols implicated in different therapies.

Evidently, fruits are the highest sources of anthocyanins, but vegetables make a large overall contribution to polyphenol intake as well. Among fruits, berries such as bilberry, blackberry, mulberry, and blackthorn have high anthocyanin contents. Both anthocyanin composition and concentration may vary among different varieties of berries, with variations arising from different factors such as environmental or climatic conditions. For example, wild, unripened varieties provide a source of variation and may influence their potential bioactivity. Environmental and agronomic conditions lead to inter-genotype variation. In blueberry, certain genotypes accumulate a higher proportion of acylated anthocyanins, which makes them different than other genotypes. This characteristic has a biological relevance because acylated anthocyanins improve color tone and stability in juices. However, reports suggested that they had a lower bioavailability than non-acylated anthocyanins. Strawberry and blackberry samples represent basic anthocyanin profiles, with pelargonidin*-3-O-*glucoside being dominant in the strawberries, and cyanidin*-3-O-*glucoside in the blackberries [113,114]. High anthocyanin content was reported in chokeberry by several studies, where cyanidin*-3-O-*galactoside was the major anthocyanin identified [115,116,117]. Cyanidin derivates were also identified in raspberry [118], mulberry [119,120], elderberry [117,121], redcurrant [122] and blackcurrant [123], along with the delphinidin derivates [124]. All those compounds identified in fruits and vegetables and their reported levels are summarized in Table 2 and Table 3, respectively.

**Table 2:** Rich anthocyanin sources of fruits and berries.

Source	Major Anthocyanin Reported	Conc. mg/100 g FW	Conc. mg/100 g DW	Conc. mg/100 mL	Ref.
Chokeberry (*Aronia melanocarpa*)	Cyanidin-3-O-galactoside		900	NR	[125]
	Cyanidin-3-O-galactoside	36.7	NR	NR	[126]
	Cyanidin-3-O-galactoside Cyanidin-3-O-arabinoside		NR	8.63	[127]
	Cyanidin-3-O-galactoside	51.3	NR	NR	[128]
	Cyanidin-3-O-galactoside	NR	NR	NR	[129]
	Cyanidin-3-O-galactoside	93.3	NR	NR	[115]
	Cyanidin-3-O-galactoside	906.9	NR	NR	[130]
	Cyanidin-3-O-galactoside	248.24	NR	NR	[117]
	Cyanidin-3-O-galactoside	403	NR	NR	[131]
	Cyanidin-3-O-galactoside	NR	8286.4	NR	[132]
	Cyanidin-3-O-galactoside	NR	NR	30.1	[133]
	Cyanidin-3-O-galactoside	NR	40	NR	[134]
	Cyanidin-3-O-galactoside	NR	627	NR	[135]
	Cyanidin-3-O-galactoside	NR	798.08	NR	[126]
Cranberries *(Vaccinium macrocarpon)*	Peonidin-3-O-galactoside	NR		75.26	[136]
	Peonidin-3-O-galactoside	NR	255	NR	[137]
	Cyanidin-3-O-arabinose	NR	588	NR	[138]
	Peonidin-3-O-glucoside	NR	NR	20.40	[139]
	Cyanidin-3-O-galactoside	15.7	NR	NR	[117]
Bilberry *(Vaccinium spp.)*	Delphinidin-3-O-glucoside	47.7	NR	NR	[140]
	Delphinidin-3-O-glucoside	NR	1761	NR	[141]
	Delphinidin-3-O-glucoside	NR	NR	57.41	[139]
	Cyanidin-3-O-glucoside	NR	NR	25.68	[127]
	Delphinidin-3-O-galactoside	177.97	NR	NR	[117]
Blueberry *(Vaccinium spp.)*	Maldivin-3-O-galactoside	101.88	NR	NR	[16]
	Maldivin-3-O-galactoside	194	NR	NR	[142]
	Maldivin-3-O-galactoside	178	NR	NR	[143]
	Cyanidin-3-O-glucoside	282	NR	NR	[144]
	Petunidin-3-O-glucoside	77.5	NR	NR	[118]
	Maldivin-3-O-galactoside, Delphinidin-3-O-galactoside	259.2	NR	NR	[145]
	Delphinidin-3-O-glucoside		NR	143.90 ± 1.56	[124]
	Delphinidin-3-O-galactoside	55.37 ± 26.2	NR	NR	[117]
	Maldivin-3-O-hexoside	NR	1218	NR	[146]
	Maldivin-3-O-galactoside Maldivin-3-O-glucoside	36.24 ± 0.6	NR	NR	[147]
	Cyanidin glycosides	424.2	NR	NR	[17]
	Delphinidin-3-O-glucoside	NR	1435	NR	[1]
	Maldivin-3-O-galactoside	32.8 ± 18.9	NR	NR	[148]
	Delphinidin-3-O-glucoside	32893 ± 2.47	NR	NR	[149]
	Maldivin glycosides		790.7 ± 19	NR	[150]
	Maldivin-3-O-galactoside	286.4 ± 37.97	NR	NR	[151]
	Delphinidin-3-O-glucoside	0.172	NR	NR	[152]
Blackberry *(Rubus spp.)*	Cyanidin-3-O-glucoside	NR	NR	17.28 ± 0.0088	[119]
	Cyanidin-3-O-glucoside	148.9 ± 69	NR	NR	[117]
	Cyanidin-3-O-glucoside	NR	606	NR	[153]
	Cyanidin-3-O-rutinoside	NR	NR	3.735	[154]
	Cyanidin-3-O-glucoside	NR	710 ± 0.02	NR	[155]
	Cyanidin-3-O-glucoside	124.3		NR	[114]
	Delphinidin-3-O-glucoside	647.0 ± 19.2		NR	[118]
	Cyanidin-3-O-glucoside	NR	811.85 ± 2.76	NR	[156]
Blackcurrant *(Ribes nigrum)*	Cyanidin-3-O-glucoside	6.599	NR	NR	[157]
	Cyanidin-3-O-glucoside	NR	NR	NR	[158]
	Delphinidin-3-O-rutinoside	27.85 ± 16.0	NR	NR	[117]
	Delphinidin-3-O-rutinoside	2.653 ± 1.82	NR	NR	[152]
	Cyanidin-3-O-rutinoside Delphinidin-3-O-rutinoside	NR	NR	8.94	[127]
	Delphinidin-3-O-rutinoside	NR	NR	140.75 ± 1.77	[134]
	Delphinidin-3-O-rutinoside	NR	NR	10.163	[154]
	Delphinidin-3-O-glucoside	644 ± 113		NR	[132]
Blackthorn (*Prunus spinose)*	Peonidin-3-O-rutinoside	NR	0.034 ± 0.03	NR	[159]
	Cyanidin-3-O-rutinoside	NR	NR	NR	[160]
	Cyanidin-3-O-glucoside	128.648 ± 116.07	NR	NR	[161]
Redcurrant (*Ribes rubrum*)	Cyanidin-3-O-glucoside	1.697	NR	NR	[157]
	Cyanidin-3-O-xylosylrutinoside	6.85 ± 2.8	NR	NR	[117]
	Cyanidin-3-O-rutinoside		NR	NR	[158]
	Cyanidin-3-O-xylosyl-rutinoside	104 ± 1.6	NR	NR	[122]
Elderberry (*Sambucus spp*.)	Cyanidin-3-O-glucoside		NR	0.3738 ± 0.147	[131]
	Cyanidin-3-O-glucoside	NR	NR	NR	[162]
	Cyanidin-3-O-glucoside Cyanidin-3-O-sambubioside	NR	NR	NR	[163]
	Cyanidin-3-O-glucoside	132.17 ± 131.9	NR	NR	[117]
Strawberry *(Fragaria spp.)*	Pelargonidin-3-O-glucoside		NR	15.13	[139]
	Pelargonidin-3-O-glucoside	61.11 ± 0.13	NR	NR	[164]
	Pelargonidin-3-O-glucoside	52.22 ± 46.4	NR	NR	[117]
	Pelargonidin-3-O-glucoside	57.9 ± 3.4	NR	NR	[165]
	Pelargonidin-3-O-glucoside		736.98 ± 178.9	NR	[166]
	Pelargonidin-3-O-glucoside	18.581	NR	NR	[167]
	Pelargonidin-3-O-glucoside	407.8 ± 16.8	NR	NR	[118]
	Pelargonidin-3-O-glucoside	21.31 ± 1.11	NR	NR	[132]
	Pelargonidin-3-O-glucoside	NR	NR	20.1	[168]
	Pelargonidin-3-O-glucoside	NR	107	NR	[165]
	Pelargonidin-3-O-glucoside	33.27	NR	NR	[123]
Mulberry *(Morus spp).*	Cyanidin-3-O-glucoside	NR	669 ± 34	NR	[130]
	Cyanidin-3-O-glucoside	NR	NR	49.2 ± 0.0099	[119]
	Cyanidin-3-O-glucoside	156.1 ± 42.3	NR	NR	[117]
	Cyanidin-3-O-glucoside	1.543 ± 0.06	NR	NR	[152]
Sour Cherry *(Prunus cerasus L)*	Cyanidin-3-O-glucosyl-rutinoside	1269.2 ± 23.3	NR	NR	[169]
	Cyanidin-3-O-glucosyl-rutinoside	NR	NR	NR	[170]
	Cyanidin-3-O-glucosyl-rutinoside	NR	372.84 ± 1.67	NR	[171]
	Cyanidin-3-O-glucosyl-rutinoside	39.02	NR	NR	[172]
	Cyanidin-3-O-glucosyl-rutinoside	59.75 ± 5.06	NR	NR	[173]
	Cyanidin-3-O-glucosyl-rutinoside	NR	NR	NR	[174]
	Cyanidin-3-O-glucosyl-rutinoside	NR	NR	NR	[175]
	Cyanidin-3-O-glucosyl-rutinoside	NR	NR	73.67	[139]
Raspberries *(Rubus idaeus)*	Cyanidin-3-O-rutinoside	NR	NR	NR	[176]
	Cyanidin-3-O-rutinoside	NR	NR	NR	[158]
	Cyanidin-3-O-sophoroside	30.56 ± 33.7	NR	NR	[117]
	Cyanidin-3-O-sophoroside	NR	233	NR	[153]
	Pelargonidin-3-O-glucoside	0.313 ± 0.01	NR	NR	[152]
	Cyanidin-3-O-sophoroside		NR	8.098	[154]
	Cyanidin-3-O-glucoside Petunidin-3-O-glucoside	133.9 ± 8.4	NR	NR	[118]
Plums (*Prunus* spp.)	Peonidin-3-O-rutinoside	NR	0.034 ± 0.03	NR	[173]
Lingonberries *(Vaccinium vitis-idaea)*	Cyanidin-3-O-glucoside	NR	NR	60.5 ± 0.054	[129]
	Cyanidin-3-O-galactoside	34.86 ± 21.5	NR	NR	[117]
Rosehip (*Rosa spp*.)	Cyanidin-3-O-glucoside	NR	0.0068	NR	[159]
	Cyanidin-3-O-glucoside	NR	0.92 ± 2.6	NR	[177]
Pomegranate (*Punica granatum*)	Cyanidin-3,5-O-diglucoside	NR	NR	NR	[178]
	Cyanidin-3,5-O-diglucoside	NR	NR	NR	[179]
	Delphinidin-3,5-O-diglucoside	NR	NR	NR	[180]
	Cyanidin-3,5-O-diglucoside	NR	NR	1.471 ± 0.32	[181]
	Delphinidin-3,5-O-diglucoside	NR	NR	NR	[182]
	Cyanidin-3,5-O-diglucoside	NR	NR	NR	[183]
	Pelargodin-3,5-O-diglucoside	17.9 ± 7.9	NR	NR	[184]
	Cyanidin-3-O-glucoside	43.99 ± 4.67	NR	NR	[185]
	Cyanidin-3-O-monoglucoside	NR	NR	NR	[186]
	Cyanidin-3-O-glucoside	NR	85 ± 0.02	NR	[187]
	Cyanidin-3,5-O-diglucoside	NR		NR	[188]
	Cyanidin-3,5-O-diglucoside	NR	75.78 ± 3.78	NR	[189]
	Cyanidin-3-O-glucoside	NR	NR	2.816 ± 0.56	[190]
	Cyanidin-3-O-glucoside	NR	NR	NR	[191]
	Cyanidin-3-O-glucoside	NR	NR	NR	[192]
	Malvidin-3-O-glucoside	NR	117 ± 4	NR	[193]
Figs (*Ficus carica*)	Cyanidin-3-O-rutinoside	1.191 ±6.33	NR	NR	[194]
	Cyanidin-3-O-rutinoside		NR	NR	[195]
	Cyanidin-3-O-rutinoside	4.82	NR	NR	[196]
Gooseberry (Ribes uva-crispa)	Cyanidin-3-O-glucoside	37.79 ± 38.1	NR	NR	[117]
	Delphinidin-3-O-rutinoside	61.19	NR	NR	[197]
	Cyanidin-3-O-glucoside	0.957 ± 1.66	NR	NR	[157]
	Cyanidin-3-O-glucoside	37.79	NR	NR	[117]
Acai (*Euterpe oleracea*)	Cyanidin-3-O-rutinoside	NR	106.7 ± 125.95	NR	[198]
	Cyanidin-3-O-glucoside	57 ± 0.39	NR	NR	[199]
	Cyanidin-3-O-glucoside		NR	NR	[200]
Acerola (*Malpighia emarginata*)	Cyanidin-3-O-rhamnoside, Pelargonidin-3-O-rhamnoside	12 ± 0.03	NR	NR	[201]
Maqui (*Aristotelia chilensis*)	Delphinidin-3-O-glucoside	715 ± 0.12	NR	NR	[202]
	Delphinidin-3-O-glucoside		4235 ± 0.08	NR	[203]
	Cyanidin-3-O-sambubioside-5-O-glucoside+ Cyanidin-diglucoside	2610	NR	NR	[204]
	Delphinidin-3-O-glucoside-5-O-glucoside	NR	1278	NR	[205]
	Delphinidin-3-O-glucoside	789 ± 0.14	NR	NR	[206]
Blood orange (*Citrus × sinensis*)	Cyanidin-3-O-(6″-malonyl glucoside)		NR	1.20 ± 0.02	[207]
Red apples	Cyanidin-3-O-galactoside	73.94 ± 31.7	NR	NR	[208]
	Cyanidin-3-O-galactoside Cyanidin-3-O-glucoside	NR	NR	NR	[209]
	Cyanidin-3-O-galactoside	21.32	NR	NR	[210]
	Cyanidin-3-O-galactoside	NR	NR	NR	[211]
Dogberry (*Cornus mas*)	Cyanidin-3-O-rutinozit chloride	NR	NR	342	[212]
	Cyanidin-3-O-galactoside	123.5 ± 19.7	NR	NR	[213]
	Peonidin-3-O-glucoside	103.37 ± 5.77	NR	NR	[214]
	Cyanidin-3-O-galactoside	104.66	NR	NR	[215]
	Pelargonidin-3-O-glucoside	NR	1403	NR	[216]
	Pelargonidin-3-O-glucoside	NR	NR	38 ± 0.052	[131]

**Table 3:** Rich anthocyanin sources of vegetables.

Source	Major Anthocyanin	Conc. mg/100 g FW	Conc. mg/100 g DW	Conc. mg/100 mL	Ref.
Native Andean Potatoes (*Solanum tuberosum, stenotonum, phureja and chaucha*)	Petunidin-3-coumaroylrutinoside-5-glucoside Pelargonidin-3-coumaroylrutinoside-5- glucoside	NR	NR	NR	[217]
Red onion (*Allium cepa*)	Cyanidin-3-(6”-malonylglucoside)	3.012 ± 1.62	NR	NR	[218]
	Cyanidin-3-(6”-malonyl)-glucopyranoside	29.99 ± 1.19	NR	NR	[156]
	Peonidin-3-O-glucoside	NR	0.19	NR	[219]
Red cabbage (*Brassica oleracea var. capitata f. rubra*)	Cyanidin-3,5-O-diglucoside	NR	232	NR	[220]
	Cyanidin-3,5-O-diglucoside	NR	NR	NR	[221]
	Cyanidin-3,5-O-diglucoside	NR	629 ± 0.25	NR	[222]
	Cyanidin-3,5-O-diglucoside	NR	588.44 ± 146.5	NR	[223]
	Cy 3-(feruloyl)diglucoside-5-glucoside	34.28 ± 1.60	NR	NR	[224]
	Cyanidin-3,5-O-diglucoside	NR	630 ± 0.09	NR	[225]
	Cyanidin-3,5-O-diglucoside	NR	NR	NR	[226]
	Cyanidin-3,5-O-diglucoside	NR	NR	NR	[227]
	Cyanidin-3-(sinapoyl)-O-diglucoside-5-O-glucoside	NR	NR	NR	[228]
	Cyanidin-3,5-O-diglucoside	NR	NR	NR	[229]
	Cyanidin-3-coumaroyl-dihexoside-5-hexoside	23.93 ± 1.02	NR	NR	[230]
	Cyanidin-3-(sinapoyl)-O-diglucoside-5-O-glucoside	NR	NR	NR	[231]
	Cyanidin derivates	NR	73.1 ± 203	NR	[232]
Purple corn (*Zea mays indurata*)	Cyanidin-3-O-glucoside	NR		NR	[233]
	Cyanidin-3-O-glucoside	NR	38.035 ± 3.39	NR	[234]
	Cyanidin-3-O-glucoside	NR		NR	[235]
	Cyanidin-3-O-glucoside	NR	83.45 ± 11.44	NR	[236]
	Cyanidin-3-O-glucoside	NR	350	NR	[237]
	Cyanidin-3-(6”-malonylglucoside)	NR	4000 ± 0.3	NR	[238]
	Cyanidin-3-O-glucoside	NR	140.69 ± 68.92	NR	[239]
	Cyanidin-3-O-glucoside	NR	3.081	NR	[240]
	Cyanidin-3-O-glucoside	NR		NR	[241]
Purple carrot (*Daucus carota subsp. sativus*)	Cyanidin-3-xylosyl(feruloylglucosyl)-galactoside	NR	1986 ± 0.36	NR	[242]
	Cyanidin-3-O-glucoside	290	NR	NR	[243]
	Cyanidin-3-xylosyl(feruloylglucosyl)galactoside	82.2 ± 0.14	NR	NR	[244]
	Cyanidin-3-xylosyl(feruloylglucosyl)galactoside		NR	NR	[245]
Radicchio (*Cichorium intybus*)	Cyanidin-3,5-di-O-(600-O-malonyl)-glucoside		NR	NR	[246]
	Cyanidin-3-O-(600-malonyl)-glucoside		NR	NR	[247]
	Cyanidin-3- O -(6”- O -malonyl)-glucoside	54.9	NR	NR	[248]
	Cyanidin-3-O-(6”-O-malonyl)-glucoside		NR	NR	[249]
	Cyanidin-3-O-(6”-Omalonyl)- glucoside	51.15 ± 23.5	NR	NR	[250]
Purple asparagus (*Asparagus officinalis*)	Cyanidin-3-O-rutinoside	3.34 ± 5.28	NR	NR	[251]
Purple kale (*Brassica oleracea*)	Cyanidin-3-(sinapoyl)diglucoside-5- glucoside	NR	NR	NR	[252]
Rhubarb (*Rheum rhabarbarum*)	Cyanidin-3-O-glucoside Cyanidin-3-O-rutinoside	NR	341.1 ± 41.6	NR	[8]
Red radish (*Raphanus raphanistrum subsp. sativus*)	Pelargonidin-3-O-(6-O-p-coumaroyl-2-O-feruloyl)-sophoroside-5-O-(6-O-malonyl)-glucoside	NR	NR	NR	[253]
	Cyanidin-3-O-sophoroside-5-O-glucoside	NR	NR	NR	[254]
	Pelargonidin-3-diglucoside-5 (malonyl)-glucoside	NR	NR	NR	[255]
Black beans (*Phaseolus vulgaris*)	Petunidin-3-O-glucoside	206	NR	NR	[256]
	Cyanidin-3-O-glucoside	NR	NR	NR	[257]
Black rice (*Oryza sativa*)	Cyanidin-3-O-glucoside	123.9 ± 31	NR	NR	[258]
	Cyanidin-3-O-glucoside	NR	140.4 ± 336	NR	[259]
	Cyanidin-3-O-glucoside	NR	41692 ± 0.36	NR	[260]
	Delphinidin-3-O-galactoside	NR	74	NR	[261]
	Cyanidin-3-O-glucoside	NR	NR	278	[262]
	Cyanidin-3-O-glucoside	NR	NR	NR	[263]
Kohlrabi (*Brassica oleracea Gongylodes Group*)	Cyanidin-3,5-O-diglucoside	NR	NR	NR	[221]
	Cyanidin-3-(caffeoyl) p-coumaroyl (sinapoyl) diglucoside-5-glucoside	NR	302 ± 0.21	NR	[264]
	Cyanidin-3-(sinapoyl)-diglucoside-5-glucoside	NR	2.3	NR	[265]
	Cyanidin-3-(feruloyl) diglucoside-5-glucoside	NR	30 ± 0.01	NR	[266]
Eggplant (*Solanum melongena*)	Delphinidin-3-glucoside-5-(coumaryl) dirhamnoside	NR	110	NR	[267]
	Delphinidin-3-O-rutinoside	4810	NR	NR	[268]
	Malvidin 3-(p-coumaroyl)rhamnoside(glucoside)-5-glucoside		202.6 ± 0.286	NR	[269]
	Malvidin-3-rutinoside-5-glucoside	NR	NR	NR	[270]
Artichoke (*Cynara cardunculus*)	Cyanidin-3-(6′-malonylglucoside)	NR	124 ± 0.04	NR	[271]
Purple sweet potato (*Ipomoea batatas*)	Peo-3-caffeoyl-feruloylsoph-5-glucoside	NR	83.8 ± 0.4	NR	[272]
	Cyanidin-3-caffeoyl-p-hydroxybenzoyl sophoroside- 5-glucoside	NR		NR	[273]
	Peonidin 3-caffeoyl-p-hydroxybenzoyl-sophoroside-5-glucoside	NR	714 ± 0.28	NR	[274]
	Peonidin-3-(6”-caffeoyl-6”‘-p-hydroxybenzoylsoph)-5-glucoside	NR	68.4	NR	[264]
	Peonidin-3-caffeoyl-p-hydroxybenzoyl sophoroside-5-glucoside	NR		NR	[248]
	Peonidin-3-(caffeoylferuloyl sophoroside)-5-glucoside	NR	730.3 ± 99.1	NR	[275]
	Peonidin-3-caffeoyl-feruloyl sophoroside-5-glucoside	NR		NR	[276]
	Cyanidin-3-(6”- caffeoyl-feruloyl sophoroside)-5-glcucoside	NR	455.08	NR	[277]
	Peonidin-3-(6”caffeoyl-6”‘p-hydroxybenzoyl sophoroside)-5-glucoside	NR		NR	[101]

## 5. Anthocyanins’ Potential for Health Benefits

Anthocyanins have been known as therapeutic agents in traditional medicine, which indicates their protective effects against various diseases in humans [5]. For instance, anthocyanins from *Hibiscus sp.* have been used effectively in folk medicines as a remedy for liver dysfunctions and hypertension [278]. Similarly, anthocyanins from *Vaccinum sp.* have been used in the treatments of visual disorders and microbial infections [279]. Despite their well-known therapeutic effects, conclusive studies regarding the pharmacological properties of anthocyanins have only been recently studied [16,32,280].

Recent in vivo studies have suggested the anthocyanins’ potential to protect against liver dysfunction, improve eyesight, provide anti-inflammatory and antimicrobial effects, decrease the level of blood pressure, and provide anti-proliferative effects on tumor cells [52] as well as prevent obesity and diabetes [281]. Epidemiological studies have shown that including rich sources of anthocyanins in daily diet is correlated with reducing the risk of many chronic diseases such as hyperlipidemia, cardiovascular diseases, Alzheimer’s disease, and various types of cancer, including breast, colon and ovarian cancer [282,283]. The anti-inflammatory effects are notable, considering inflammation plays a key role in the carcinogenesis in animals, and probably in humans [284]. Moreover, anthocyanins have been shown to protect from DNA cleavage, estrogenic activity, enzyme inhibition, lipid peroxidation, decreasing capillary permeability, and membrane strengthening [285,286,287].

Currently, the potential health benefits of anthocyanins are strongly related to their antioxidant activity [288]. Due to their phenolic structure, anthocyanins are potent antioxidants in vitro. They are capable of scavenging the free-radicals, which are responsible for increased oxidative stress [289].

Reactive oxygen species are typically generated in the body. However, if they are overly produced, they can lead to cellular damage, which further leads to inflammations, cardiovascular diseases, cancer, and premature aging [290]. The antioxidant activity of these pigments is of particular importance at neutral pH because pH in the human body is generally neutral, except in the stomach [289]. As reported previously [291], the ability of free-radical scavenging is particularly due to anthocyanin chalcones and quinoidal bases with a double bond conjugated to the keto group. Moreover, the glycosylated B-ring in the structure of anthocyanins are highly effective regarding their antioxidant activity, where ortho-hydroxylation and methoxylation significantly increase the antioxidant activity [292]. Several in vitro studies have reported the antioxidant effect of anthocyanins when conducted on the colon [293,294], endothelial [295], liver [296], breast [226,239], leukemic [123] and keratinocytes [297], cervical [280] and melanoma [124] cell lines. In these studies, anthocyanins have shown to exhibit multiple anticarcinogenic effects from scavenging reactive oxygen species to reducing cell proliferation, from stimulating the expression of Phase II detoxification enzymes to inhibiting mutagenesis by environmental toxins and carcinogenesis, among others. Anthocyanins possess the hydroxyl groups in both position 3 of ring C and also in the 3′, 4′, and 5′ positions in ring B in their chemical structure. This pattern provides powerful antioxidant activity. Generally, anthocyanin aglycones are better at radical scavenging compared to anthocyanins. However, their radical scavenging activity diminishes as sugar fraction enhances [28].

Anthocyanins were also shown to possess strong metal chelators and reducing agents. In a study, purified and standardized ethanolic extracts of 10 varieties of elderberry fruits, which were high in cyanidin aglycone, were examined for iron and copper chelating and reducing activities [298]. The authors reported that cyanidin, cyanidin-3-glucoside as standards and all the extracts of elderberry fruits could chelate iron and also reduced both iron and copper. However, the standards and the extracts were found to be poor in chelating copper. Among the varieties of elderberries, variations in metal chelating and reducing properties were also observed.

The role of anthocyanins in preventing cardiovascular diseases is strongly related to oxidative stress protection. The oxidative stress induces a cellular redox imbalance, which often occurs in some types of cancer, and it is related to oncogene stimulation. Oxidative damage leads to genetic mutations that represent the first step of mutagenesis, carcinogenesis, and aging [55]. That being said, the latest studies have emphasized the potential health benefits of active compounds from different natural sources and suggested some useful applications for controlling the pathogenesis of chronic diseases caused by oxidative stress.

Anthocyanins have been shown to exhibit a variety of biological activities leading to their anticarcinogenic properties depending on the different substituents on their B-rings. Based on the relevant studies in the literature, including the use of various cancer cell lines, animal models and human clinical trials, anthocyanins have been shown to have an antitumor or anticancer role throughout the tumorigenesis and carcinogenesis, from the initial stage of tumorigenesis to cancer formation and cancer development stages. These roles have been extensively explained elsewhere [11]. The authors in the previous review paper grouped the activities of anthocyanins as an antioxidant, anti-inflammation, anti-mutagenesis for the initial stage of tumorigenesis; differentiation induction, inhibiting cellular transformation, cell proliferation, and signaling pathways from blocking signal transduction and regulating the expression of anti-oncogenes and relevant proteins in the cancer formation stage; inducing apoptosis of tumor cells, inhibiting angiogenesis of tumors in the cancer development stage and reducing the expression of cell adhesion and influencing the expression of components for extracellular matrix degradation to inhibit the invasion and metastasis of tumors. The authors also reported that the anthocyanins could reverse the multidrug resistance of cancer. However, the metabolism of anthocyanins is still uncertain [11]. In this context, we are presenting an overview of published data about anthocyanin potential in human health, focusing mainly on melanoma treatment, along with other types of cancers taking both in vivo and in vitro studies into consideration.

## 6. Anthocyanins Involvement in Cancer Prevention

Cancer is one of the most controversial and frequently debated topics, and it is one of the leading causes of global mortality. Today, many studies focus on finding novel methods for cancer prevention. Over the years, various pharmaceuticals have been extensively used in cancer therapy. Chemotherapy, for instance, is one of the conventional treatments commonly recommended to patients with metastatic tumors. However, nearly all anticancer drugs do not target rapidly proliferating cells. Therefore, they have the potential of life-threatening side effects, often limiting their efficient use due to the high risk of alteration of healthy tissues and organs. This may lead to severe consequences, such as blood cell aplasia as a consequence of chemotherapy, secondary immunodeficiency, deterioration of the detoxify cation function of the liver, and many others. Thus, developing novel anticancer agents with a lower level of toxicity remains a priority for the worldwide scientific community. The association of a diet rich in fruits and vegetables with a lower occurrence of several types of human cancer is a largely accepted concept. It has been reported that phytochemicals such as polyphenols from fruits and vegetables are able to induce apoptosis in cells or cell arrest in cancerous cells, while showing little or no toxic effects [299]. The organism could reap the benefits of these properties only if there is a combination of phytochemicals included in our diet since pure or limited compounds are not enough for maximum efficiency [20]. The detrimental effects of conventional cytostatic towards healthy tissues can be attenuated with the help of auxiliary therapy. Phenolic compounds such as anthocyanins play an important role in antitumor therapy via bioactive compounds. Previous in vivo, in vitro or clinical studies, have reported that anthocyanins have the ability to decrease the proliferation of cancerous cells and to inhibit the development of tumors [293,294,296,300,301,302]. Some studies found that anthocyanins and their aglycones were able to inhibit the growth of tumor cells, whilst the growth of healthy cells was little or not affected at all [303,304]. Nevertheless, their anticancer effects may vary among different cancer cell lines. Antimetastasis agents have been recognized as a novel category of cancer chemopreventive agents. Reports have suggested that cyanidin and pelargonidin sourced from blackberry or strawberry exert chemopreventive activity [305,306,307].

Recently, the interest of the worldwide scientific community on the biochemical aspects and biological effects of anthocyanins has substantially expanded, in response to the evidence demonstrating not only their broad therapeutic potential but also those that may have beneficial anticarcinogenic effects. In fact, various studies have proved their anticarcinogenic and anti-proliferative effects, as well as their role as chemopreventive agents. Several anthocyanin-based extracts from different plant sources have been shown to exhibit anti-proliferative activity towards multiple cancer cell types in vitro [124,300]. Cell proliferation was found to be inhibited by the ability of anthocyanins to block various stages of the cell cycle via effects on cell cycle regulator proteins [308].

The process of programmed cell death, also known as “apoptosis”, plays a significant role in maintaining the homeostasis among normal cell populations. Apoptosis potently suffers malfunctions in cancer cells; thus, some of the most efficient chemopreventive agents have been designed to induce apoptosis in premalignant and malignant cells [1]. Anthocyanin-rich extracts from berries and grapes have been found to exhibit proapoptotic effects when tested on various cancer cell lines [305]. Likewise, anthocyanins extracted from purple-fleshed sweet potato have been shown to have beneficial effects against colorectal cancer via apoptotic mechanisms. The treatment significantly inhibited aberrant crypt foci formation in the colons of female CF-1 mice, which has been correlated with a higher expression of apoptotic caspase-3 in the colon mucosal epithelial cells [309]. Similarly, a recent study investigated the effect of purple sweet potato anthocyanins on the proliferation of bladder cancer cell line BIU87. The data obtained showed that purple sweet potato anthocyanins could inhibit the bladder cancer BIU87 cell growth via apoptosis [250].

Another study has also demonstrated that anthocyanin extract of purple potato, through TNF-related apoptosis-inducing ligand, stimulates the maturation of acute myeloid leukemia cells. Moreover, anthocyanins from wine sources have been reported to have an anti-invasive property in human hepatoma Hep3B cells [310]. The effects of anthocyanins were also investigated on human lung cancer [21]. In this study, the potential of anthocyanins from fruits of *Vitits coignetiae Pulliat* to inhibit cell proliferation, invasion, and angiogenesis in human lung cancer A549 cells was investigated. These results bring evidence that the extract might have anticancer effects on human lung cancer. All these studies provide clear information about all chemopreventive, proapoptotic, and anti-proliferative effects of anthocyanins against various types of cancer. Moreover, these results demonstrate that anthocyanins are effective in vitro for many types of cancer.

Among all these types of cancer, melanoma is the one that is less studied; however, it is one of the most aggressive types. In this context, our next section will be focused on in vitro studies examining anthocyanins’ role in melanoma treatment.

## 7. Anthocyanins as Potential Agents for Melanoma Prevention

**Figure 4:**
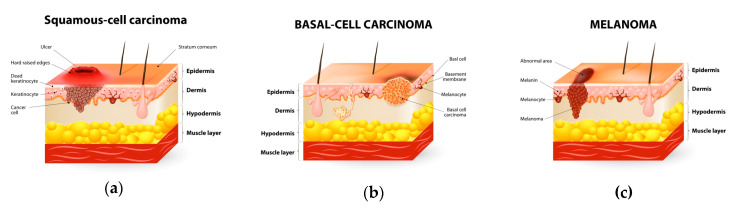
The illustrations of the types of skin cancer, namely: (**a**) Squamous cell carcinoma; (**b**) Basal cell cancer and (**c**) Melanoma.

Skin cancer can be defined as the relentless division of cancer cells in the skin. There are three different types of skin cancers. The most common cancer in humans is basal cell carcinoma (BCC), which belongs to a non-melanoma subset, together with squamous cell carcinoma (SCC). The third major category of skin cancer is represented by melanoma (Figure 4). Merkel cell tumors and dermatofibrosarcoma protuberans are also considered as cancer types. Melanoma represents a type of skin cancer that starts in melanocytes. Melanocytes are the cells that can become a melanoma, and they are specialized pigment-producing cells found in both the basal layer of the epidermis and in the eye [14]. The number of patients diagnosed with melanoma has been increasing faster than the number of patients diagnosed with any other type of cancer [311].

Over 200,000 of new cases of melanoma and 50,000 of related deaths were registered worldwide in 2012, making the melanoma ranked at 15th among common cancers worldwide [312]. Currently, 132,000 melanoma skin cancers occur globally each year, one in every three cancer cases being diagnosed as skin cancer (WHO). The incidence of cutaneous melanoma varies markedly among regions; these variations being strongly correlated to the racial skin phenotype and the level of sun exposure. Unlike other cancers, melanoma mainly affects young and middle-aged individuals, with the median age at diagnosis being 57 years [313]. Analyzing the statistics related to sex, women cases occur more frequently in younger aged categories, while men prevail from the age of 55 onwards [314]. Most of the patients are diagnosed at an early stage. For these patients, surgical excision is curative in the majority of cases [315]; however, there is the risk of relapse.

On the other hand, approximately 10% of melanoma cases are diagnosed when the disease has already reached an advanced stage, when they are often already metastatic [313]. Therefore, cutaneous melanoma represents one of the most aggressive forms of skin cancer due to its incredible ability to spread and grow [313]. Malignant tumors are composed of cancer cells, having the ability to invade nearby normal tissues. These types of tumors entail the degradation of the extracellular matrix, cell metastasis, and proliferation. It is biologically the most aggressive form of skin cancer because it is spreading rapidly to other body parts, usually in five phases, each of which is characterized by specific molecular aberrations [316,317] such as the overexpression of proteolytic enzyme activity, such as matrix metalloproteinases, as well as the detaching and migration of tumor cells into the bloodstream or lymph nodes from where they can spread to any other tissue or organ [318].

Over the past decade, the strategies to improve melanoma treatment were notably advanced. Researchers have been focused on a deeper understanding of the essential mechanisms involved in melanoma development and its biology. Due to the increasing incidence of melanoma in the United States, most of the studies aimed to develop more efficient drugs with lower toxicity in order to prevent and treat melanoma. Experimental approaches have used agents that modify tumor growth by inducing terminal differentiation [319].

The most vital role of the skin is to protect the body from environmental aggressions. The skin has epidermal units which are composed of a melanocyte surrounded by keratinocytes and regulated by a closed paracrine system. The epidermal units are responsible for melanogenesis. Melanogenesis is defined as a complex process that involves different stages through melanin synthesis and distribution [320]. Besides determining skin color and some phenotypic aspects, melanin plays an essential role in protecting the skin against ultraviolet light. Increased levels of melanin in the epidermis lead to hyperpigmentation, which can cause severe skin damages such as melasma, lentigo, or post-inflammatory hyperpigmentation [321]. Although melanocytes represent only 1% of total epidermal cells, they are responsible for melanin synthesis. These cells can be found in the basal layer of the epidermis as well in the choroidal layer of the eye [322]. Melanin is transferred to keratinocytes, where it acts as protection against solar radiation [318]. Although the knowledge of melanocyte biology has made remarkable progress, there is still much to be clarified. Investigating the mechanism of melanogenesis is critical for developing photoprotective measures, which reduce photoaging and photocarcinogenesis. Although there are no 100% curative treatments for melanoma yet, efficient results can be achieved by early diagnosis. Prevention has the main role in detecting melanoma in order to prevent more advanced stages.

The most important external factor for melanoma skin cancer development is exposure to UV radiation. It is well-known that excessive exposure to UV radiation leads to premature skin aging, hyperkeratosis, and premalignant lesions [323]. The increased risk of melanoma development due to sun exposure is directly associated with the UV level, particularly the UVB spectrum (280–320 nm) [324]. Exposure to these types of radiations has been demonstrated to initiate inflammatory pathways and oxidative damage in the epidermis and dermis, leading to an increased risk of skin cancers, including risk for the development of melanoma [325]. Moreover, UVA radiation (320–400 nm), even though less powerful than UVB radiation, has the ability to penetrate deeper into the skin layers [300].

The increasingly high number of patients diagnosed with skin cancer has led to high demand for finding active compounds that can efficiently fight against UVR-induced skin damage [325]. Other factors critical for the development of melanoma are the number of congenital and acquired melanocytic nevi along with the genetic susceptibility and family history [326,327,328]. Contrary to belief, no other environmental factors, such as tobacco/smoke addition, have been associated with melanoma [311]. Currently, early diagnosis is the main option for curing this disease, while prevention strategies are mandatory in spotting the disease at earlier and more curable stages [329].

Including biochemotherapy and biological agents into the classical treatment has been shown to be more effective than traditional treatments, which are based only on chemotherapy alone [330,331,332,333,334]. Most anticancer treatments are originated from natural resources, including marine, microbial, and botanical sources [335]. Natural supplements and a diet rich in antioxidants for the purpose of the complementary medication are the current topics of related research. Several studies have demonstrated that flavonoids and anthocyanins are suitable candidates for the prevention of the adverse effects of UV radiation due to their UV absorbing property and antioxidant properties. Table 4 summarizes the positive biological effects of anthocyanins in vivo. There are different available cell lines to create animal models of cancers. In the case of melanoma, there are different melanoma cell lines, for instance, melanoma B16F10 (metastatic), B16F0 (non-metastatic), which all derived from C57Bl/6 mice; B16-F0 being the parent cell line. The B16F10 model is very aggressive at the biological level, much more than the B16F0, which remains close to the parental B16 one and grows slowly.

Anthocyanins can act [336] as immune response stimulators, or they can induce gene suppression or block oxidative damage to DNA [337,338]. One study demonstrated that blackberry anthocyanins could reduce UV mediated oxidative injury in skin keratinocytes [339]. Bog blueberry anthocyanins have been found to possess protective effects against UVB-induced skin photoaging. This action occurs by blocking collagen destruction and inflammatory responses via transcriptional mechanisms of NF-κB (nuclear factor-kappaB) along with MAPK signaling [340]. The photoprotective properties of strawberry were previously shown in human dermal fibroblasts. One of the effective and reliable inhibitory solutions is natural bioactive compounds, such as anthocyanins, which are known for having an inhibitory effect on melanogenesis in human melanocytes [336].

In plants, anthocyanins gather in the vacuoles of epidermal cells. These pigments were associated with the protective effects against solar radiation a long time ago. Considering the photoprotective effects of anthocyanins in plants, researchers have started from the hypothesis that similar effects may be shown on human skin [325]. The photoprotective role of the anthocyanins is increased by the flavylium cation form of anthocyanins, strongly absorbing UV light [341]. Anthocyanins have strong absorptions in both visible and ultraviolet regions in the electromagnetic spectrum, particularly in the 505–550 and 280–320 nm ranges [342]. Anthocyanins sourced from purple sweet potato were included in the formulation of a cosmetic product at a concentration of 0.61 mg/100 g of cream, and they were able to absorb almost half of the UV radiation (46%) in vitro [343]. This has revealed that even low concentrations of anthocyanins may limit the amount of UVB radiation interacting with the epidermis, indicating the benefits of anthocyanins in preventing UV-induced skin damage. Another study has reported the protective effects of anthocyanins from black soybean coats both in in vitro keratinocytes and in vivo hairless mice [344]. A clinical study also stated that a formulation including anthocyanins and glutathione were found to be efficient in lowering the skin erythema caused by radiation therapy in breast cancer patients [345]. However, more studies are required to determine the most effective dose and response in clinical trials, as well as to clarify the effectiveness of anthocyanins as photoprotective compounds.

The studies have been focused on the antioxidant and detoxifying properties of anthocyanins as well as their effects on decreasing the rate of cell replication and thus controlling tumor growth [346]. Reports have shown that phenolic fractions inhibit melanogenic activity and decrease the development of melanoma cells [346]. This data suggest anthocyanins’ potential as therapeutic agents regarding the treatment of human melanoma. Many studies, both in vitro and in vivo, have been done on demonstrating the effects that anthocyanins, derived from various plant sources, have on different melanoma cell lines. It is well known that B16–F10 melanoma cells are a highly invasive metastatic cell line. A study reported that anthocyanin-rich fraction of blueberries has the ability to inhibit proliferation, stimulate apoptosis, and increase lactate dehydrogenase leakage activity in B16-F10 melanoma murine cells, after 24 h of treatment [16].

Another study proved that mulberry anthocyanin extract prevents atherosclerosis and inhibits melanoma metastasis [31]. The applied treatment with mulberry anthocyanin extracts on B16-F1 cells revealed within Western blotting assay that the expression levels of Ras, phosphoinositide 3-kinase (PI3K), phospho-Akt, and nuclear factor kappa B (NF-κB) were reduced after 24 h. Moreover, it was suggested that mulberry anthocyanin extracts could mediate B16-F1 cell metastasis by reduction of MMP-2 and MMP-9 activities involving the suppression of the Ras/ PI3K signaling pathway. They also carried out experiments in vivo, of B16-F1 melanoma cells being injected into the right groin of the C57BL/6 mice, and the mice were fed with the same mulberry anthocyanin extracts. The immunohistochemistry stain results and also hematoxylin-eosin stain showed that the applied treatment inhibited the metastasis of B16- F1 cells in vivo.

**Table 4:** Anthocyanins from different food matrices and their positive biological effects against melanoma.

Cell Line	Anthocyanins Sources	Conc.	Biological Effect	Ref.
C1 41	Black Raspberry	0–100 μg/mL	↓tumor progression	[347]
B16-F1	Mulberry	0–5 mg/mL	↓cell proliferation ↓viability	[31]
B16	Cyanidin-3-α-*O*-rhamnoside Pelargodin-3- α-*O*-rhamnoside	0–20 μg/mL	↓melanin content ✓skin-lightening ↓tyrosinase activity	[25]
MeOMEZlan-a mouse melanocytes	Red wine	4–500 mg/L	↓melanogenic activity ↓tyrosinase activity	[346]
TVM-A12	Cyanidin*-3-O-*β-glucopyranoside	5/10 µM	↓cell proliferation induced ✓morphological differentiation	[319]
B16-F1	Mulberry	0–3 mg/mL	↓cells proliferation	[31]
B16	*Liriope platyphylla*	0–500 μg/mL	↓tyrosinase activity ↓melanin content	[348]
B16-F10	Blueberry	0–800 μg/mL	✓antioxidant activity ↓cells proliferation ✓apoptosis ↑LDH activity	[19]
A375	Hibiscus sabdariffa Linn.	5–50 mg/mL	↓ melanin synthesis ↓tyrosinase activity	[349]
B16-F10	Strawberry		↓cell proliferation	[55]
B16-F10	Blueberry and blackcurrant juices	0–500 µg/mL	↓cell proliferation	[21]
B16-F10	Delphinidin	10 μg/mL	↓endothelial cells proliferation	[295]
B-16	Fructus Sorbi acupariae	5 mL/kg	✓antitumor activity ✓antimetastatic activity ↑stromal progenitor cells	[350]
A375, A549	*Rubus fairholmianus*	10–40 µg/mL	↓cell proliferation ↓viability ↑cytotoxicity ✓apoptosis	[351]
A375, B16-F10	Chokeberry, red grape	0–400 µg/mL	↓cell proliferation ↑oxidative stress biomarkers ↓Δψ	[21]
B16-F10	Blueberry	0–800 μg/mL	↓viability ↓cell proliferation ✓blocked cell cycle G0/G1 phase ✓apoptosis	[20]
A375	Houttuynia cordata Thunb	25–200 μg/mL	↓viability ↓cell proliferation ✓apoptosis	[352]
B16-F10	Elderberries		↓cell proliferation ↑LDH activity	[20]
B16-F10	*Dendrobium*	0–120 μg/mL	↓cell viability ↑melanin inhibition ↑enzyme inhibition	[353]
WM35	*Origanum vulgare*	0–4 μg/mL	↓cell viability	[22]
B16-F1	*Hibiscus sabdariffa calyx*	0–1 mg/mL	↓cell growth ↓ migration ↓tube formation ↓MMP–2/–9 and VEGF ↓migration and angiogenesis	[354]

↑- increase activity, ↓-decrease activity, ✓- poses this actitivy.

Anthocyanins are the most important type of flavonoids and widely distributed phenolic compounds in strawberry. A study investigated the antiproliferative activity of anthocyanin-rich strawberry extracts on the B16–F10 murine melanoma cell line [51], which provided preliminary data for the ability of the extract to inhibit the growth and induce differentiation in a melanoma cancer cell line. Treatment of melanoma cells with the extract has affected several parameters of melanoma cells, proving the antitumor activities on the highly metastatic B16-F10 murine melanoma cell line. Therefore, the report has clearly demonstrated a remarkable inhibition of cell proliferation in the treated B16-F10 cells (about 30% after 48 h and by about 27% after 72 h compared to the control application. Trypan Blue exclusion test has also revealed that the strawberry extract caused no cell injury, and the toxicity level was lower than 5% in B16-F10 cells. The same authors lately conducted another study and reported the anti-proliferative activity of extracts from cell suspensions (strawberry, strawberry tree, blackberry, and red raspberry) on murine melanoma cells. The extracts containing anthocyanins were found to reduce cell proliferation (ranging from 30% to 38% compared to the control) [355].

A further study investigated the effects of delphinidin on tumor development [295]. In vivo, delphinidin was shown to significantly decrease melanoma-induced tumor growth, while the data obtained after the in vitro experiment exhibited a decrease of endothelial cell proliferation. The influence of cyanidin-3-*O*-β-glucopyranoside (C-3-G) on TVM-A12 human melanoma cell line was investigated. C-3-G is widely distributed among plants and included in the diet [341]. The study investigated the effects of C-3-G on cell proliferation and morphology as well as melanin synthesis. C-3-G treatment was shown to affect cell proliferation and induce morphological differentiation. Furthermore, melanin formation and melanosome maturation were enhanced. Another study reported the photo-chemopreventive effect of delphinidin against UVB-induced biomarkers of skin cancer development [356] when tested on SKH-1 hairless mice. The results of the treatment suggested that delphinidin was able to inhibit UVB-mediated oxidative stress and reduced DNA damage, indicating that this anthocyanin may protect the cells from UVB-induced apoptosis.

Mulberry anthocyanins were investigated for their potential antimetastatic activity [350]. The data obtained suggested that mulberry anthocyanins had strong anticancer effects and inhibited the metastasis ability of B16-F1 cells. The antimetastatic effect of these compounds was also apparent in a C57BL/6 mice model.

Anthocyanins possess high antioxidant activity; however, they are highly susceptible to the impact of environmental factors. Therefore, encapsulating anthocyanin into liposomes was found to effectively stabilize these molecules. A study investigated the inhibitory effects of liposome-capsulated anthocyanin (LCA) isolated from *Hibiscus sabadariffa Linn*. on melanoma development in human A357 melanocytes [349]. The study demonstrated that anthocyanin with liposome encapsulation increased the stabilization of anthocyanin and the inhibition of melanogenesis.

Several studies have shown that anthocyanins exhibit antiproliferative and proapoptotic effects. Recently, a study using anthocyanin-enriched extracts (AEE) was obtained from elderberries, and the extract was used for the treatment of B16-F10 murine melanoma cells. After the treatment, AEE inhibited cell proliferation in a concentration-dependent manner and increased LDH activity. Moreover, AEE induced apoptosis of melanoma cells, confirmed by dual staining AO/EB and TUNEL assay. The data obtained in this study indicate that elderberry-derived anthocyanins may be utilized in skin cancer therapy due to their high anthocyanin content [20].

Blueberries are known for their high anthocyanin content, which has been shown to be involved in different cancer-associated processes. In a recent study, the chemopreventive potential of anthocyanin extracts from blueberry was investigated on the B16-F10 melanoma cell line. After the treatment, both anthocyanin and anthocyanidin extracts inhibited the viability and proliferation of melanoma cells. In this study, anthocyanin extract from the same berries was tested as well on murine melanoma cells, where it was found to exhibit higher cytotoxicity than anthocyanin extracts. Both extracts blocked cell cycle progression at the G0/G1 phase at a concentration lower than 400 and 200 µg/mL. The induction of apoptosis was observed using flow cytometric analysis. The data obtained shows that anthocyanin and anthocyanidin extracts might be used in order to treat skin cancer and in topical applications [17]

Anthocyanins and their aglycones have been proved to exhibit various biological effects, including anticarcinogenic potential on different types of cancer cell lines. In our latest study, the phytochemical content of nine different berry samples was investigated using liquid chromatography. The study focused on the effects of selected chokeberry and red grape anthocyanin extracts (C-ARE and RG-ARE) on melanoma cell lines. After the treatment with the extract, both C-ARE and RG-ARE anthocyanins reduced cell proliferation, increased oxidative stress biomarkers, and decreased mitochondrial membrane potential, without a negative effect on the normal cells. At the same time, RG-ARE was more effective in the treatment due to its five different aglycones. We concluded that C-ARE and RG-ARE anthocyanins might be effective in reducing cell proliferation and increasing the level of oxidative stress in cancer cells [18].

## 8. Anthocyanins as Functional Ingredients in Cosmetics

Along with increasing consumer preferences for products with functional ingredients, the cosmetic products embedded with polyphenols have also become more trendy. Thanks to healthy aspects of secondary plant metabolites, functional ingredients with such properties have recently become prevalent not only in cosmetics but also in pharmaceutical industries [357]. Accordingly, the number and variety of products with beneficial ingredients, also known as cosmeceuticals, are growing to meet the demand [357,358]. Based on the studies in the literature, anthocyanins were previously shown to constrain some of the reactions causing photoaging and skin diseases. The concept of selecting highly stable anthocyanins with previously reported health benefits and including them in topical applications can lead to desired properties observed in cosmetic products. However, the studies on evaluating the applications of active compounds in such products have been limited up to today. Although the benefit of such compounds to the skin was previously shown and accepted, not much is known as to penetration of these compounds to the stratum corneum (SC). Among the three layers of the skin (epidermis, dermis, and hypodermis), with a typical thickness of 10–20 μm, SC forms the outermost layer of the epidermis [359]. It is the main barrier of the skin, avoiding the water and electrolyte losses [360]. In order to be effective, anthocyanins applied in topical formulas should be released, in order to reach and overcome the SC so that it can reach the layers underneath [361]. The rate and the kinetics at which active components are released from the product and penetrate the skin depend on the molecular properties of the specific compound, including molecular weight and lipophilicity, together with the characteristics of the vehicle [362].

On the other hand, drugs can penetrate the skin through hair follicles, interfollicular sites, corneocytes, and lipid bilayer membranes [363]. The approaches for enhancing the permeation of the compounds into the skin in topical or “trans-dermal” drug delivery is a currently investigated topic for drug developers. Although the addition of some complexes may lead to better penetration of the active compounds [364], the use of the additional compounds is not commonly used since they irritate the skin at high concentrations [362].

There have been both in vitro and in vivo percutaneous studies conducted regarding the efficacy of the topical formulations on drug delivery and skin barrier properties. In one study, lipsticks with elderberry or red radish included in the formulations were evaluated for skin permeation and stratum corneum penetration in a porcine ear model [365] and yielded encouraging results. Briefly, the lipstick formulations containing anthocyanins showed their properties to act as antioxidants, and also, they were demonstrated to be effective on tyrosinase inhibition. The positive results were obtained at physiologically relevant concentrations taking the common lipstick usage within the United States. In another study, black soybean extracts were used on human skin, and the allergic response was evaluated. The results have revealed that the use of black soybean extract caused no allergic reaction on human patch trials and encouraged the potential use of such extracts high in anthocyanins as additives in cosmetic formulations for purposes such as anti-aging and whitening [366]. Another study evaluated the anthocyanins extracted from grapes and blackcurrants for skin-lightening goals by including them in the topical delivery of protein-rich formulations [367]. The authors reported no antimicrobial effect upon the use of the formula. This also supports the inclusion of anthocyanins in topical formulations.

## 9. Conclusions and Future Perspectives

The attention on anthocyanin research regarding human health benefits has expanded from diabetes and cardiovascular diseases to cancer in the last years. Extensive evidence has been provided about the multiple positive health effects of these bioactive compounds and their presumed mechanisms of action. There is a new trend to investigate how dietary polyphenols, especially anthocyanins, are implicated in the prevention or evolution of different chronic diseases. Rich sources of anthocyanins are represented by a large variety of fruits, vegetables, as well as various edible flowers. Phytochemical and pharmacological studies have validated the therapeutic uses of edible plants, and many reports on phytochemical composition have demonstrated that berries are rich sources of anthocyanins being effective in ameliorating various degenerative diseases. Additionally, if certain foods with particular groups of anthocyanins, as in the case of berries, are more beneficial than other fruits or vegetables is not clear yet. Anthocyanin extracts were shown to provide antioxidant effects, although the exact mechanism by which anthocyanins prevent the development of some diseases remains to be elucidated.

Moreover, the bioavailability and dosage of the extracts is yet another concern that has to be focused on. In this work, previous studies on anthocyanin content of common foods from the human daily diet have been reviewed in order to find out their current status. This review focuses on the chemopreventive activity of anthocyanins, and the most recent studies on these compounds regarding melanoma treatment are presented. Many studies suggested that anthocyanins from various plant sources exert anticarcinogenic and anti-proliferative effects and are able to reduce the damaging effects of reactive oxygen species. Recently, antimetastatic agents were defined as a new class of cancer chemopreventive agents, and several studies have demonstrated that cyanidin and pelargonidin obtained from different types of berries, exhibited chemopreventive and chemotherapeutic activity. Furthermore, the blueberry anthocyanin and anthocyanidin extracts are potential raw materials for the production of antitumor health foods and medicines.

In summary, this systematic review provides further support that anthocyanins reduce the risk of cancer; however, without doubt, in vivo animal studies or human clinical trials are more convincing in this area. Until a more precise conclusion can be drawn, we would recommend that a healthy dietary intake should include anthocyanin-rich sources as well as a varied diet of fruit and vegetables rich in other bioactive compounds. For future perspectives, further studies would be necessary in order to clarify the possible mechanisms and to evaluate the bioavailability of anthocyanins before they can be used extensively in clinical applications to reduce tumor and cancer risk.

## References

[1] Zhou Y., Zheng J., Li Y., Xu D.-P., Li S., Chen Y.-M., Li H.-B. (2016). Natural polyphenols for prevention and treatment of cancer. Nutrients.

[2] Lage N.N., Layosa M.A.A., Arbizu S., Chew B.P., Pedrosa M.L., Mertens-Talcott S., Talcott S., Noratto G.D. (2020). Dark sweet cherry (prunus avium) phenolics enriched in anthocyanins exhibit enhanced activity against the most aggressive breast cancer subtypes without toxicity to normal breast cells. J. Funct. Foods.

[3] Koch W. (2019). Dietary polyphenols-important non-nutrients in the prevention of chronic noncommunicable diseases. A systematic review. Nutrients.

[4] Isah T. (2019). Stress and defense responses in plant secondary metabolites production. Biol. Res..

[5] Lila M.A. (2004). Anthocyanins and human health: An in vitro investigative approach. J. Biomed. Biotechnol..

[6] Scalbert A., Johnson I.T., Saltmarsh M. (2005). Polyphenols: Antioxidants and beyond. Am. J. Clin. Nutr..

[7] Wallace T.C., Giusti M.M. (2015). Anthocyanins. Adv. Nutr..

[8] Takeoka G.R., Dao L., Harden L., Pantoja A., Kuhl J.C. (2013). Antioxidant activity, phenolic and anthocyanin contents of various rhubarb (rheum spp.) varieties. Food Sci. Technol..

[9] Bimpilas A., Panagopoulou M., Tsimogiannis D., Oreopoulou V. (2016). Anthocyanin copigmentation and color of wine: The effect of naturally obtained hydroxycinnamic acids as cofactors. Food Chem..

[10] Khoo H.E., Azlan A. (2017). Anthocyanidins and anthocyanins: Colored pigments as food, pharmaceutical ingredients, and the potential health benefits. Food Nutr. Res..

[11] Lin B.W., Gong C.C., Song H.F., Cui Y.Y. (2017). Effects of anthocyanins on the prevention and treatment of cancer. Br. J. Pharmacol..

[12] Zhao X., Feng P., He W., Du X., Chen C., Suo L., Liang M., Zhang N., Na A., Zhang Y. (2019). The prevention and inhibition effect of anthocyanins on colorectal cancer. Curr. Pharm. Des..

[13] Rager E.L., Bridgeford E.P., Ollila D.W. (2005). Cutaneous melanoma: Update on prevention, screening, diagnosis, and treatment. Am. Fam. Physician.

[14] Hurst E.A., Harbour J.W., Cornelius L.A. (2003). Ocular melanoma: A review and the relationship to cutaneous melanoma. Arch. Dermatol..

[15] Wiecker T.S., Luther H., Buettner P., Bauer J., Garbe C. (2003). Moderate sun exposure and nevus counts in parents are associated with development of melanocytic nevi in childhood: A risk factor study in 1,812 kindergarten children. Cancer.

[16] Bunea A., Rugina D., Sconta Z., Pop R.M., Pintea A., Socaciu C., Tabaran F., Grootaert C., Struijs K., VanCamp J. (2013). Anthocyanin determination in blueberry extracts from various cultivars and their antiproliferative and apoptotic properties in b16-f10 metastatic murine melanoma cells. Phytochemistry.

[17] Wang E., Liu Y., Xu C., Liu J. (2017). Antiproliferative and proapoptotic activities of anthocyanin and anthocyanidin extracts from blueberry fruits on b16-f10 melanoma cells. Food Nutr. Res..

[18] Diaconeasa Z., Ayvaz H., Rugina D., Leopold L., Stanila A., Socaciu C., Tabaran F., Luput L., Mada D.C., Pintea A. (2017). Melanoma inhibition by anthocyanins is associated with the reduction of oxidative stress biomarkers and changes in mitochondrial membrane potential. Plant Foods Hum. Nutr..

[19] Benedec D., Oniga I., Cuibus F., Sevastre B., Stiufiuc G., Duma M., Hanganu D., Iacovita C., Stiufiuc R., Lucaciu C.M. (2018). Origanum vulgare mediated green synthesis of biocompatible gold nanoparticles simultaneously possessing plasmonic, antioxidant and antimicrobial properties. Int. J. Nanomed..

[20] Rugină D., Hanganu D., Diaconeasa Z., Tăbăran F., Coman C., Leopold L., Bunea A., Pintea A. (2017). Antiproliferative and apoptotic potential of cyanidin-based anthocyanins on melanoma cells. Int. J. Mol. Sci..

[21] Lu J.N., Panchanathan R., Lee W.S., Kim H.J., Kim D.H., Choi Y.H., Kim G.S., Shin S.C., Hong S.C. (2017). Anthocyanins from the fruit of vitis coignetiae pulliat inhibit tnf-augmented cancer proliferation, migration, and invasion in a549 cells. Asian Pac. J. Cancer Prev. Apjcp.

[22] Hanamura T., Uchida E., Aoki H. (2008). Skin-lightening effect of a polyphenol extract from acerola (Malpighia emarginata DC.) fruit on uv-induced pigmentation. Biosci. Biotechnol. Biochem..

[23] Mazza G., Francis F.J. (1995). Anthocyanins in grapes and grape products. Crit. Rev. Food Sci. Nutr..

[24] Felgines C., Talavera S., Texier O., Gil-Izquierdo A., Lamaison J.L., Remesy C. (2005). Blackberry anthocyanins are mainly recovered from urine as methylated and glucuronidated conjugates in humans. J. Agric. Food Chem..

[25] Andersen O.M., Markham K.R., Andersen O.M., Markham K.R. (2006). The Anthocyanins. Flavonoids Chemistry, Biochemistry and Applications.

[26] Hsu Y.H., Tagami T., Matsunaga K., Okuyama M., Suzuki T., Noda N., Suzuki M., Shimura H. (2017). Functional characterization of udp-rhamnose-dependent rhamnosyltransferase involved in anthocyanin modification, a key enzyme determining blue coloration in lobelia erinus. Plant J..

[27] Vicente Dragano N.R., Castro Marques A.Y., Mauricio D. (2016). Chapter 11—Native Fruits, Anthocyanins in Nutraceuticals, and the Insulin Receptor/Insulin Receptor Substrate-1/Akt/Forkhead Box Protein Pathway. Molecular Nutrition and Diabetes.

[28] Wang L.-S.S., Gary D. (2008). Anthocyanins and their role in cancer prevention. Cancer Lett..

[29] Trouillas P., Sancho-García J.C., De Freitas V., Gierschner J., Otyepka M., Dangles O. (2016). Stabilizing and modulating color by copigmentation: Insights from theory and experiment. Chem. Rev..

[30] Boulton R. (2001). The copigmentation of anthocyanins and its role in the color of red wine: A critical review. Am. J. Enol. Vitic..

[31] Huang H.-P., Shih Y.-W., Chang Y.-C., Hung C.-N., Wang C.-J. (2008). Chemoinhibitory effect of mulberry anthocyanins on melanoma metastasis involved in the ras/pi3k pathway. J. Agric. Food Chem..

[32] Tsuda T. (2012). Dietary anthocyanin-rich plants: Biochemical basis and recent progress in health benefits studies. Mol. Nutr. Food Res..

[33] Yeh C.T., Yen G.C. (2005). Induction of apoptosis by the anthocyanidins through regulation of bcl-2 gene and activation of c-jun n-terminal kinase cascade in hepatoma cells. J. Agric. Food Chem..

[34] Katsube N., Iwashita K., Tsushida T., Yamaki K., Kobori M. (2003). Induction of apoptosis in cancer cells by bilberry (vaccinium myrtillus) and the anthocyanins. J. Agric. Food Chem.

[35] Borges G., Roowi S., Rouanet J.M., Duthie G.G., Lean M.E., Crozier A. (2007). The bioavailability of raspberry anthocyanins and ellagitannins in rats. Mol. Nutr. Food Res..

[36] Felgines C., Texier O., Besson C., Fraisse D., Lamaison J.L., Rémésy C. (2002). Blackberry anthocyanins are slightly bioavailable in rats. J. Nutr..

[37] Felgines C., Talavéra S., Gonthier M.P., Texier O., Scalbert A., Lamaison J.L., Rémésy C. (2003). Strawberry anthocyanins are recovered in urine as glucuro- and sulfoconjugates in humans. J. Nutr..

[38] Ichiyanagi T., Shida Y., Rahman M.M., Hatano Y., Konishi T. (2006). Bioavailability and tissue distribution of anthocyanins in bilberry (vaccinium myrtillus l.) extract in rats. J. Agric. Food Chem..

[39] Marczylo T.H., Cooke D., Brown K., Steward W.P., Gescher A.J. (2009). Pharmacokinetics and metabolism of the putative cancer chemopreventive agent cyanidin-3-glucoside in mice. Cancer Chemother. Pharmacol..

[40] Matsumoto H., Ichiyanagi T., Iida H., Ito K., Tsuda T., Hirayama M., Konishi T. (2006). Ingested delphinidin-3-rutinoside is primarily excreted to urine as the intact form and to bile as the methylated form in rats. J. Agric. Food Chem..

[41] Fang J. (2014). Bioavailability of anthocyanins. Drug Metab. Rev..

[42] Czank C., Cassidy A., Zhang Q., Morrison D.J., Preston T., Kroon P.A., Botting N.P., Kay C.D. (2013). Human metabolism and elimination of the anthocyanin, cyanidin-3-glucoside: A (13)c-tracer study. Am. J. Clin. Nutr..

[43] Passamonti S., Vrhovsek U., Vanzo A., Mattivi F. (2003). The stomach as a site for anthocyanins absorption from food. Febs Lett..

[44] Passamonti S., Vrhovsek U., Vanzo A., Mattivi F. (2005). Fast access of some grape pigments to the brain. J. Agric. Food Chem..

[45] Vanzo A., Terdoslavich M., Brandoni A., Torres A.M., Vrhovsek U., Passamonti S. (2008). Uptake of grape anthocyanins into the rat kidney and the involvement of bilitranslocase. Mol. Nutr. Food Res..

[46] Tian Q., Giusti M.M., Stoner G.D., Schwartz S.J. (2006). Urinary excretion of black raspberry (rubus occidentalis) anthocyanins and their metabolites. J. Agric. Food Chem..

[47] Wu X., Pittman H.E., McKay S., Prior R.L. (2005). Aglycones and sugar moieties alter anthocyanin absorption and metabolism after berry consumption in weanling pigs. J. Nutr..

[48] de Ferrars R.M., Czank C., Zhang Q., Botting N.P., Kroon P.A., Cassidy A., Kay C.D. (2014). The pharmacokinetics of anthocyanins and their metabolites in humans. Br. J. Pharmacol..

[49] Keppler K., Humpf H.U. (2005). Metabolism of anthocyanins and their phenolic degradation products by the intestinal microflora. Bioorganic Med. Chem..

[50] Talavéra S., Felgines C., Texier O., Besson C., Gil-Izquierdo A., Lamaison J.L., Rémésy C. (2005). Anthocyanin metabolism in rats and their distribution to digestive area, kidney, and brain. J. Aagric. Food Chem..

[51] Forni C., Braglia R., Mulinacci N., Urbani A., Ronci M., Gismondi A., Tabolacci C., Provenzano B., Lentini A., Beninati S. (2014). Antineoplastic activity of strawberry (fragaria × ananassa duch.) crude extracts on b16-f10 melanoma cells. Mol. Biosyst..

[52] Konczak I., Zhang W. (2004). Anthocyanins—More than nature’s colours. J. Biomed. Biotechnol..

[53] Hogan S., Chung H., Zhang L., Li J., Lee Y., Dai Y., Zhou K. (2010). Antiproliferative and antioxidant properties of anthocyanin-rich extract from açai. Food Chem..

[54] Jaakola L. (2013). New insights into the regulation of anthocyanin biosynthesis in fruits. Trends Plant Sci..

[55] Fang J. (2015). Classification of fruits based on anthocyanin types and relevance to their health effects. Nutrition.

[56] Escribano-Bailón M.T., Santos-Buelga C., Rivas-Gonzalo J.C. (2004). Anthocyanins in cereals. J. Chromatogr. A.

[57] Kruger M.J., Davies N., Myburgh K.H., Lecour S. (2014). Proanthocyanidins, anthocyanins and cardiovascular diseases. Food Res. Int..

[58] Hertog M.G., Feskens E.J., Hollman P.C., Katan M.B., Kromhout D. (1993). Dietary antioxidant flavonoids and risk of coronary heart disease: The zutphen elderly study. Lancet.

[59] Skibola C.F., Smith M.T. (2000). Potential health impacts of excessive flavonoid intake. Free Radic Biol Med..

[60] Markakis P. (1982). Anthocyanins as Food Colors.

[61] Burton-Freeman B., Sandhu A., Edirisinghe I., Gupta R.C. (2016). Chapter 35—Anthocyanins. Nutraceuticals.

[62] Garzón G.A., Manns D.C., Riedl K., Schwartz S.J., Padilla-Zakour O. (2015). Identification of phenolic compounds in petals of nasturtium flowers (tropaeolum majus) by high-performance liquid chromatography coupled to mass spectrometry and determination of oxygen radical absorbance capacity (orac). J. Agric. Food Chem..

[63] Park K.-I., Hoshino A., Saito N., Tatsuzawa F. (2014). Anthocyanins in the flowers of ipomoea tricolor cav. (convolvulaceae). Biochem. Syst. Ecol..

[64] Phippen W.B., Simon J.E. (1998). Anthocyanins in basil (ocimum basilicum l.). J. Agric. Food Chem..

[65] Szymanowska U., Zlotek U., Karas M., Baraniak B. (2015). Anti-inflammatory and antioxidative activity of anthocyanins from purple basil leaves induced by selected abiotic elicitors. Food Chem..

[66] Goupy P., Vian M.A., Chemat F., Caris-Veyrat C. (2013). Identification and quantification of flavonols, anthocyanins and lutein diesters in tepals of crocus sativus by ultra performance liquid chromatography coupled to diode array and ion trap mass spectrometry detections. Ind. Crop. Prod..

[67] Nørbæk R., Kondo T. (1999). Further anthocyanins from flowers of crocus antalyensis (iridaceae). Phytochemistry.

[68] Kazuma K., Kogawa K., Noda N., Kato N., Suzuki M. (2004). Identification of delphinidin 3-o-(6’’-o-malonyl)-beta-glucoside-3’-o-beta-glucoside, a postulated intermediate in the biosynthesis of ternatin c5 in the blue petals of clitoria ternatea (butterfly pea). Chem. Biodivers..

[69] Nair V., Bang W.Y., Schreckinger E., Andarwulan N., Cisneros-Zevallos L. (2015). Protective role of ternatin anthocyanins and quercetin glycosides from butterfly pea (clitoria ternatea leguminosae) blue flower petals against lipopolysaccharide (lps)-induced inflammation in macrophage cells. J. Agric. Food Chem..

[70] Grajeda-Iglesias C., Salas E., Barouh N., Barea B., Figueroa-Espinoza M.C. (2017). Lipophilization and ms characterization of the main anthocyanins purified from hibiscus flowers. Food Chem..

[71] Hashimoto M., Suzuki T., Iwashina T. (2011). New acylated anthocyanins and other flavonoids from the red flowers clematis cultivars. Sage J..

[72] Jia N., Shu Q.-Y., Wang L.-S., Du H., Xu Y.-J., Liu Z.-A. (2008). Analysis of petal anthocyanins to investigate coloration mechanism in herbaceous peony cultivars. Sci. Hortic..

[73] Byamukama R., Namukobe J., Jordheim M., Andersen Ø.M., Kiremire B.T. (2011). Anthocyanins from ornamental flowers of red frangipani, plumeria rubra. Sci. Hortic..

[74] Terahara N., Oda M., Matsui T., Osajima Y., Saito N., Toki K., Honda T. (1996). Five new anthocyanins, ternatins a3, b4, b3, b2, and d2, from clitoria ternatea flowers. J. Nat. Prod..

[75] Li J.-B., Hashimoto F., Shimizu K., Sakata Y. (2008). Anthocyanins from red flowers of camellia cultivar ‘dalicha’. Phytochemistry.

[76] Li J.B., Hashimoto F., Shimizu K., Sakata Y. (2009). A new acylated anthocyanin from the red flowers of camellia hongkongensis and characterization of anthocyanins in the section camellia species. J. Integr. Plant Biol..

[77] Nørbæk R., Brandt K., Nielsen J.K., Ørgaard M., Jacobsen N. (2002). Flower pigment composition of crocus species and cultivars used for a chemotaxonomic investigation. Biochem. Syst. Ecol..

[78] Park C.H., Chae S.C., Park S.Y., Kim J.K., Kim Y.J., Chung S.O., Arasu M.V., Al-Dhabi N.A., Park S.U. (2015). Anthocyanin and carotenoid contents in different cultivars of chrysanthemum (dendranthema grandiflorum ramat.) flower. Molecules.

[79] Chen S.-M., Li C.-H., Zhu X.-R., Deng Y.-M., Sun W., Wang L.-S., Chen F.-D., Zhang Z. (2012). The identification of flavonoids and the expression of genes of anthocyanin biosynthesis in the chrysanthemum flowers. Biol. Plant..

[80] Sakaguchi K., Kitajima J., Iwashina T. (2013). Acylated delphinidin glycosides from violet and violet-blue flowers of clematis cultivars and their coloration. Nat. Prod. Commun.

[81] Nakayama M., Koshioka M., Yoshida H., Kan Y., Fukui Y., Koike A., Yamaguchi M.-a. (2000). Cyclic malyl anthocyanins in dianthus caryophyllus. Phytochemistry.

[82] Okamura M., Nakayama M., Umemoto N., Cano E.A., Hase Y., Nishizaki Y., Sasaki N., Ozeki Y. (2013). Crossbreeding of a metallic color carnation and diversification of the peculiar coloration by ion-beam irradiation. Euphytica.

[83] Fukui Y., Tanaka Y., Kusumi T., Iwashita T., Nomoto K. (2003). A rationale for the shift in colour towards blue in transgenic carnation flowers expressing the flavonoid 3′,5′-hydroxylase gene. Phytochemistry.

[84] Bloor S.J. (1998). A macrocyclic anthocyanin from red\mauve carnation flowers. Phytochemistry.

[85] Koike A., Barreira J.C., Barros L., Santos-Buelga C., Villavicencio A.L., Ferreira I.C. (2015). Edible flowers of viola tricolor l. As a new functional food: Antioxidant activity, individual phenolics and effects of gamma and electron-beam irradiation. Food Chem.

[86] Sun W., Liang L., Meng X., Li Y., Gao F., Liu X., Wang S., Gao X., Wang L. (2016). Biochemical and molecular characterization of a flavonoid 3-o-glycosyltransferase responsible for anthocyanins and flavonols biosynthesis in freesia hybrida. Front. Plant Sci..

[87] Grajeda-Iglesias C., Figueroa-Espinoza M.C., Barouh N., Baréa B., Fernandes A., de Freitas V., Salas E. (2016). Isolation and characterization of anthocyanins from hibiscus sabdariffa flowers. J. Nat. Prod..

[88] Xu W., Luo G., Yu F., Jia Q., Zheng Y., Bi X., Lei J. (2018). Characterization of anthocyanins in the hybrid progenies derived from iris dichotoma and domestica by hplc-dad-esi/ms analysis. Phytochemistry.

[89] Yabuya T., Yamaguchi M.-a., Imayama T., Katoh K., Ino I. (2002). Anthocyanin 5-o-glucosyltransferase in flowers of iris ensata. Plant Sci..

[90] Imayama T., Yoshihara N., Fukuchi-Mizutani M., Tanaka Y., Ino I., Yabuya T. (2004). Isolation and characterization of a cdna clone of udp-glucose: Anthocyanin 5-o-glucosyltransferase in iris hollandica. Plant Sci..

[91] Saito N., Toki K., Kuwano H., Moriyama H., Shigihara A., Honda T. (1995). Acylated cyanidin 3-rutinoside-5,3′-diglucoside from the purple-red flower of lobelia erinus. Phytochemistry.

[92] Markham K.R., Mitchell K.A., Boase M.R. (1997). Malvidin-3-o-glucoside-5-o-(6-acetylglucoside) and its colour manifestation in ‘johnson’s blue’ and other ‘blue’ geraniums. Phytochemistry.

[93] Jia N., Shu Q., Wang D., Wang L., Liu Z., Ren H., Xu Y., Tian D., Tilt K.M. (2008). Identification and characterization of anthocyanins by high-performance liquid chromatography–electrospray ionization–mass spectrometry in herbaceous peony species. J. Amer. Soc. Hort. Sci..

[94] Du H., Wu J., Ji K.-X., Zeng Q.-Y., Bhuiya M.-W., Su S., Shu Q.-Y., Ren H.-X., Liu Z.-A., Wang L.-S. (2015). Methylation mediated by an anthocyanin, o-methyltransferase, is involved in purple flower coloration in paeonia. J. Exp. Bot..

[95] Prinsi B., Negri A.S., Quattrocchio F.M., Koes R.E., Espen L. (2016). Proteomics of red and white corolla limbs in petunia reveals a novel function of the anthocyanin regulator anthocyanin1 in determining flower longevity. J. Proteom..

[96] Ando T., Tatsuzawa F., Saito N., Takahashi M., Tsunashima Y., Numajir H., Watanabe H., Kokubun H., Hara R., Seki H. (2000). Differences in the floral anthocyanin content of red petunias and petunia exserta. Phytochemistry.

[97] Griesbach R.J., Stehmann J.R., Meyer F. (1999). Anthocyanins in the “red” flowers of petunia exserta. Phytochemistry.

[98] Saito R., Fukuta N., Ohmiya A., Itoh Y., Ozeki Y., Kuchitsu K., Nakayama M. (2006). Regulation of anthocyanin biosynthesis involved in the formation of marginal picotee petals in petunia. Plant Sci..

[99] Zhang L., Fu Q., Zhang Y. (2011). Composition of anthocyanins in pomegranate flowers and their antioxidant activity. Food Chem..

[100] Lee J.H., Lee H.-J., Choung M.-G. (2011). Anthocyanin compositions and biological activities from the red petals of korean edible rose (rosa hybrida cv. Noblered). Food Chem..

[101] Zhang J.-L., Luo C.-L., Zhou Q., Zhang Z.-C. (2018). Isolation and identification of two major acylated anthocyanins from purple sweet potato (ipomoea batatas l. Cultivar eshu no. 8) by uplc-qtof-ms/ms and nmr. Food Sci. Technol..

[102] Navarro-González I., González-Barrio R., García-Valverde V., Bautista-Ortín A.B., Periago M.J. (2015). Nutritional composition and antioxidant capacity in edible flowers: Characterisation of phenolic compounds by hplc-dad-esi/ms(n). Int. J. Mol. Sci..

[103] Yuan Y., Ma X., Tang D., Shi Y. (2014). Comparison of anthocyanin components, expression of anthocyanin biosynthetic structural genes, and tff3′h1 sequences between tulipa fosteriana ‘Albert heijn’ and its reddish sport. Sci. Hortic..

[104] Tatsuzawa F. (2015). Differences in the floral anthocyanin content of violet–blue flowers of vinca minor l. And v. Major l. (apocynaceae). Phytochem. Lett..

[105] González-Barrio R., Periago M.J., Luna-Recio C., Garcia-Alonso F.J., Navarro-González I. (2018). Chemical composition of the edible flowers, pansy (viola wittrockiana) and snapdragon (antirrhinum majus) as new sources of bioactive compounds. Food Chem..

[106] Ge Q., Ma X. (2013). Composition and antioxidant activity of anthocyanins isolated from yunnan edible rose (an ning). Food Sci. Hum. Wellness.

[107] Fossen T., Rayyan S., Holmberg M.H., Nateland H.S., Andersen O.M. (2005). Acylated anthocyanins from leaves of oxalis triangularis. Phytochemistry.

[108] Alexandra Pazmiño-Durán E., Mónica Giusti M., Wrolstad R.E., Glória M.B.A. (2001). Anthocyanins from oxalis triangularis as potential food colorants. Food Chem..

[109] Fossen T., Rayyan S., Holmberg M.H., Nimtz M., Andersen O.M. (2007). Covalent anthocyanin-flavone dimer from leaves of oxalis triangularis. Phytochemistry.

[110] Harborne A.J. (1998). Phytochemical Methods a Guide to Modern Techniques Plant Analysis.

[111] Manach C., Williamson G., Morand C., Scalbert A., Remesy C. (2005). Bioavailability and bioefficacy of polyphenols in humans. I. Review of 97 bioavailability studies. Am. J. Clin. Nutr.

[112] Tsao R. (2010). Chemistry and biochemistry of dietary polyphenols. Nutrients.

[113] Ariza M.T., Reboredo-Rodríguez P., Cervantes L., Soria C., Martínez-Ferri E., González-Barreiro C., Cancho-Grande B., Battino M., Simal-Gándara J. (2018). Bioaccessibility and potential bioavailability of phenolic compounds from achenes as a new target for strawberry breeding programs. Food Chem..

[114] Van de Velde F., Grace M.H., Esposito D., Pirovani M.É., Lila M.A. (2016). Quantitative comparison of phytochemical profile, antioxidant, and anti-inflammatory properties of blackberry fruits adapted to argentina. J. Food Compos. Anal..

[115] Hwang S.J., Yoon W.B., Lee O.-H., Cha S.J., Kim J.D. (2014). Radical-scavenging-linked antioxidant activities of extracts from black chokeberry and blueberry cultivated in korea. Food Chem..

[116] Thi N., Hwang E.-S. (2018). Effects of black chokeberry extracts on metastasis and cell-cycle arrest in sk-hep1 human liver cancer cell line. Asian Pac. J. Trop. Biomed..

[117] Veberic R., Slatnar A., Bizjak J., Stampar F., Mikulic-Petkovsek M. (2015). Anthocyanin composition of different wild and cultivated berry species. Lwt Food Sci. Technol..

[118] Marhuenda J., Alemán M.D., Gironés-Vilaplana A., Pérez A., Caravaca G., Figueroa F., Mulero J., Zafrilla P. (2016). Phenolic composition, antioxidant activity, and in vitro availability of four different berries. J. Chem..

[119] Sang J., Ma Q., Li C.-q. (2017). Development and validation of green chromatography for the determination of anthocyanins in haskap berry, mulberry and blackberry. Anal. Methods.

[120] Tomas M., Toydemir G., Boyacioglu D., Hall R., Beekwilder J., Capanoglu E. (2015). The effects of juice processing on black mulberry antioxidants. Food Chem..

[121] Antolak H., Czyzowska A., Sakač M., Mišan A., Đuragić O., Kregiel D. (2017). Phenolic compounds contained in little-known wild fruits as antiadhesive agents against the beverage-spoiling bacteria asaia spp.. Molecules.

[122] Mattila P.H., Hellström J., Karhu S., Pihlava J.-M., Veteläinen M. (2016). High variability in flavonoid contents and composition between different north-european currant (ribes spp.) varieties. Food Chem..

[123] Feng R., Ni H.M., Wang S.Y., Tourkova I.L., Shurin M.R., Harada H., Yin X.M. (2007). Cyanidin-3-rutinoside, a natural polyphenol antioxidant, selectively kills leukemic cells by induction of oxidative stress. J. Biol. Chem..

[124] Diaconeasa Z., Leopold L., Rugina D., Ayvaz H., Socaciu C. (2015). Antiproliferative and antioxidant properties of anthocyanin rich extracts from blueberry and blackcurrant juice. Int. J. Mol. Sci..

[125] Taheri R., Connolly B.A., Brand M.H., Bolling B.W. (2013). Underutilized chokeberry (aronia melanocarpa, aronia arbutifolia, aronia prunifolia) accessions are rich sources of anthocyanins, flavonoids, hydroxycinnamic acids, and proanthocyanidins. J. Agric. Food Chem..

[126] Wojdyło A., Oszmiański J., Teleszko M., Sokół-Łętowska A. (2013). Composition and quantification of major polyphenolic compounds, antioxidant activity and colour properties of quince and mixed quince jams. Int. J. Food Sci. Nutr..

[127] Šavikin K., Zdunić G., Janković T., Gođevac D., Stanojković T., Pljevljakušić D. (2014). Berry fruit teas: Phenolic composition and cytotoxic activity. Food Res. Int..

[128] Gardana C., Ciappellano S., Marinoni L., Fachechi C., Simonetti P. (2014). Bilberry adulteration: Identification and chemical profiling of anthocyanins by different analytical methods. J. Agric. Food Chem..

[129] Worsztynowicz P., Napierała M., Białas W., Grajek W., Olkowicz M. (2014). Pancreatic α-amylase and lipase inhibitory activity of polyphenolic compounds present in the extract of black chokeberry (aronia melanocarpa l.). Process. Biochem..

[130] Rugină D., Diaconeasa Z., Coman C., Bunea A., Socaciu C., Pintea A. (2015). Chokeberry anthocyanin extract as pancreatic β-cell protectors in two models of induced oxidative stress. Oxidative Med. Cell. Longev..

[131] Vlachojannis C., Zimmermann B.F., Chrubasik-Hausmann S. (2015). Quantification of anthocyanins in elderberry and chokeberry dietary supplements. Phytother. Res. Ptr.

[132] Oszmiański J., Lachowicz S. (2016). Effect of the production of dried fruits and juice from chokeberry (aronia melanocarpa l.) on the content and antioxidative activity of bioactive compounds. Molecules.

[133] Tomić M., Ignjatović Đ., Tovilović-Kovačević G., Krstić-Milošević D., Ranković S., Popović T., Glibetić M. (2016). Reduction of anxiety-like and depression-like behaviors in rats after one month of drinking aronia melanocarpa berry juice. Food Funct..

[134] Ćujić N., Šavikin K., Janković T., Pljevljakušić D., Zdunić G., Ibrić S. (2016). Optimization of polyphenols extraction from dried chokeberry using maceration as traditional technique. Food Chem..

[135] Ćujić N., Savikin K., Miloradovic Z., Ivanov M., Vajic U.-J., Karanovic D., Grujic-Milanovic J., Jovovic D., Mihailovic-Stanojevic N. (2018). Characterization of dried chokeberry fruit extract and its chronic effects on blood pressure and oxidative stress in spontaneously hypertensive rats. J. Funct. Foods.

[136] Lee J. (2013). Proanthocyanidin a2 purification and quantification of american cranberry (vaccinium macrocarpon ait.) products. J. Funct. Foods.

[137] Hummer K., Durst R., Zee F., Atnip A., Giusti M.M. (2014). Phytochemicals in fruits of hawaiian wild cranberry relatives. J. Sci. Food Agric..

[138] Gao C., Zhao S., Yagiz Y., Gu L. (2018). Static, kinetic, and isotherm adsorption performances of macroporous adsorbent resins for recovery and enrichment of bioactive procyanidins from cranberry pomace. J. Food Sci..

[139] Díaz-García M.C., Obón J.M., Castellar M.R., Collado J., Alacid M. (2013). Quantification by uhplc of total individual polyphenols in fruit juices. Food Chem..

[140] Aaby K., Grimmer S., Holtung L. (2013). Extraction of phenolic compounds from bilberry (vaccinium myrtillus l.) press residue: Effects on phenolic composition and cell proliferation. Lwt Food Sci. Technol..

[141] Primetta A.K., Jaakola L., Ayaz F.A., Inceer H., Riihinen K.R. (2013). Anthocyanin fingerprinting for authenticity studies of bilberry (vaccinium myrtillus l.). Food Control..

[142] Buran T.J., Sandhu A.K., Li Z., Rock C.R., Yang W.W., Gu L. (2014). Adsorption/desorption characteristics and separation of anthocyanins and polyphenols from blueberries using macroporous adsorbent resins. J. Food Eng..

[143] Correa-Betanzo J., Allen-Vercoe E., McDonald J., Schroeter K., Corredig M., Paliyath G. (2014). Stability and biological activity of wild blueberry (vaccinium angustifolium) polyphenols during simulated in vitro gastrointestinal digestion. Food Chem..

[144] Paes J., Dotta R., Barbero G.F., Martínez J. (2014). Extraction of phenolic compounds and anthocyanins from blueberry (vaccinium myrtillus l.) residues using supercritical co2 and pressurized liquids. J. Supercrit. Fluids.

[145] Zhang S.-L., Deng P., Xu Y.-C., Lü S.-W., Wang J.-J. (2016). Quantification and analysis of anthocyanin and flavonoids compositions, and antioxidant activities in onions with three different colors. J. Integr. Agric..

[146] Cardenosa V., Girones-Vilaplana A., Muriel J.L., Moreno D.A., Moreno-Rojas J.M. (2016). Influence of genotype, cultivation system and irrigation regime on antioxidant capacity and selected phenolics of blueberries (vaccinium corymbosum l.). Food Chem.

[147] Figueira M.E., Oliveira M., Direito R., Rocha J., Alves P., Serra A.T., Duarte C., Bronze R., Fernandes A., Brites D. (2016). Protective effects of a blueberry extract in acute inflammation and collagen-induced arthritis in the rat. Biomed. Pharm..

[148] Li D., Li B., Ma Y., Sun X., Lin Y., Meng X. (2017). Polyphenols, anthocyanins, and flavonoids contents and the antioxidant capacity of various cultivars of highbush and half-high blueberries. J. Food Compos. Anal..

[149] Wu Y., Zhou Q., Chen X.Y., Li X., Wang Y., Zhang J.L. (2017). Comparison and screening of bioactive phenolic compounds in different blueberry cultivars: Evaluation of anti-oxidation and alpha-glucosidase inhibition effect. Food Res. Int.

[150] Zorenc Z., Veberic R., Stampar F., Koron D., Mikulic-Petkovsek M. (2017). Thermal stability of primary and secondary metabolites in highbush blueberry (vaccinium corymbosum l.) purees. Lwt Food Sci. Technol..

[151] Oh H.D., Yu D.J., Chung S.W., Chea S., Lee H.J. (2018). Abscisic acid stimulates anthocyanin accumulation in ‘jersey’ highbush blueberry fruits during ripening. Food Chem..

[152] Chen L., Xin X., Yuan Q., Su D., Liu W. (2014). Phytochemical properties and antioxidant capacities of various colored berries: Phytochemical properties and antioxidant capacities of colored berries. J. Sci. Food Agric..

[153] Blando F., Gerardi C., Renna M., Castellano S., Serio F. (2018). Characterisation of bioactive compounds in berries from plants grown under innovative photovoltaic greenhouses. J. Berry Res..

[154] Ljevar A., Ćurko N., Tomašević M., Radošević K., Srček V.G., Ganić K.K. (2016). Phenolic composition, antioxidant capacity and in vitro cytotoxicity assessment of fruit wines. Food Technol. Biotechnol..

[155] Ivanovic J., Tadic V., Dimitrijevic S., Stamenic M., Petrovic S., Zizovic I. (2014). Antioxidant properties of the anthocyanin-containing ultrasonic extract from blackberry cultivar “čačanska bestrna”. Ind. Crop. Prod..

[156] Dos Santos S.S., Rodrigues L.M., Da Costa S.C., De Cassia Bergamasco R., Madrona G.S. (2017). Microencapsulation of bioactive compounds from blackberry pomace (rubus fruticosus) by spray drying technique. Int. J. Food Eng..

[157] Laczko-Zold E., Komlosi A., Ulkei T., Fogarasi E., Croitoru M., Fulop I., Domokos E., Stefanescu R., Varga E. (2018). Extractability of polyphenols from black currant, red currant and gooseberry and their antioxidant activity. Acta Biol Hung..

[158] Feng C., Su S., Wang L., Wu J., Tang Z., Xu Y., Shu Q., Wang L. (2016). Antioxidant capacities and anthocyanin characteristics of the black–red wild berries obtained in northeast china. Food Chem..

[159] Guimaraes R., Barros L., Duenas M., Carvalho A.M., Queiroz M.J., Santos-Buelga C., Ferreira I.C. (2013). Characterisation of phenolic compounds in wild fruits from northeastern portugal. Food Chem.

[160] Velickovic J., Kostic D., Stojanovic G., Mitic S., Mitic M., Randjelovic S., Djordjevic A. (2014). Phenolic composition, antioxidant and antimicrobial activity of the extracts from prunus spinosa l. Fruit. Hem. Ind..

[161] Mikulic-Petkovsek M., Stampar F., Veberic R., Sircelj H. (2016). Wild prunus fruit species as a rich source of bioactive compounds: Wild prunus-rich source of bioactive compound. J. Food Sci..

[162] Duymuş H.G., Göger F., Başer K.H.C. (2014). In vitro antioxidant properties and anthocyanin compositions of elderberry extracts. Food Chem..

[163] Szalóki-Dorkó L., Csizmadia G., Abrankó L., Stéger-Máté M. (2015). Examination of anthocyanin content of some elderberry cultivars grown in hungary. Acta Hortic..

[164] Giampieri F., Alvarez-Suarez J.M., Mazzoni L., Forbes-Hernandez T.Y., Gasparrini M., Gonzàlez-Paramàs A.M., Santos-Buelga C., Quiles J.L., Bompadre S., Mezzetti B. (2014). An anthocyanin-rich strawberry extract protects against oxidative stress damage and improves mitochondrial functionality in human dermal fibroblasts exposed to an oxidizing agent. Food Funct..

[165] Misran A., Padmanabhan P., Sullivan J.A., Khanizadeh S., Paliyath G. (2014). Composition of phenolics and volatiles in strawberry cultivars and influence of preharvest hexanal treatment on their profiles. Can. J. Plant Sci..

[166] Fernandez-Lara R., Gordillo B., Rodriguez-Pulido F.J., Lourdes Gonzalez-Miret M., Del Villar-Martinez A.A., Davila-Ortiz G., Heredia F.J. (2015). Assessment of the differences in the phenolic composition and color characteristics of new strawberry (fragaria x ananassa duch.) cultivars by hplc-ms and imaging tristimulus colorimetry. Food Res. Int.

[167] Bursac Kovacevic D., Putnik P., Dragovic-Uzelac V., Vahcic N., Babojelic M.S., Levaj B. (2015). Influences of organically and conventionally grown strawberry cultivars on anthocyanins content and color in purees and low-sugar jams. Food Chem.

[168] Arend G.D., Adorno W.T., Rezzadori K., Di Luccio M., Chaves V.C., Reginatto F.H., Petrus J.C.C. (2017). Concentration of phenolic compounds from strawberry ( fragaria x ananassa duch ) juice by nanofiltration membrane. J. Food Eng..

[169] Kołodziejczyk K., Sójka M., Abadias M., Viñas I., Guyot S., Baron A. (2013). Polyphenol composition, antioxidant capacity, and antimicrobial activity of the extracts obtained from industrial sour cherry pomace. Ind. Crop. Prod..

[170] Yılmaz F.M., Karaaslan M., Vardin H. (2015). Optimization of extraction parameters on the isolation of phenolic compounds from sour cherry (prunus cerasus l.) pomace. J. Food Sci. Technol..

[171] Zorić Z., Pedisić S., Kovačević D.B., Ježek D., Dragović-Uzelac V. (2016). Impact of packaging material and storage conditions on polyphenol stability, colour and sensory characteristics of freeze-dried sour cherry (prunus cerasus var. Marasca). J. Food Sci. Technol..

[172] Karaaslan M., Yılmaz F.M., Karaaslan A., Vardin H. (2016). Synthesis and accumulation of anthocyanins in sour cherries during ripening in accordance with antioxidant capacity development and chalcone synthase expression. Eur. Food Res. Technol..

[173] Cao J., Jiang Q., Lin J., Li X., Sun C., Chen K. (2015). Physicochemical characterisation of four cherry species (prunus spp.) grown in china. Food Chem..

[174] Picariello G., De Vito V., Ferranti P., Paolucci M., Volpe M.G. (2016). Species- and cultivar-dependent traits of prunus avium and prunus cerasus polyphenols. J. Food Compos. Anal..

[175] Picariello G., Ferranti P., De Cunzo F., Sacco E., Volpe M.G. (2017). Polyphenol patterns to trace sweet (prunus avium) and tart (prunus cerasus) varieties in cherry jam. J. Food Sci. Technol..

[176] Kula M., Majdan M., Głód D., Krauze-Baranowska M. (2016). Phenolic composition of fruits from different cultivars of red and black raspberries grown in poland. J. Food Compos. Anal..

[177] Cunja V., Mikulic-Petkovsek M., Zupan A., Stampar F., Schmitzer V. (2015). Frost decreases content of sugars, ascorbic acid and some quercetin glycosides but stimulates selected carotenes in rosa canina hips. J. Plant Physiol.

[178] Türkyılmaz M. (2013). Anthocyanin and organic acid profiles of pomegranate (punica granatum l.) juices from registered varieties in turkey. Int. J. Food Sci. Technol..

[179] Sentandreu E., Cerdán-Calero M., Sendra J.M. (2013). Phenolic profile characterization of pomegranate (punica granatum) juice by high-performance liquid chromatography with diode array detection coupled to an electrospray ion trap mass analyzer. J. Food Compos. Anal..

[180] Gomez-Caravaca A.M., Verardo V., Toselli M., Segura-Carretero A., Fernandez-Gutierrez A., Caboni M.F. (2013). Determination of the major phenolic compounds in pomegranate juices by hplc-dad-esi-ms. J. Agric. Food Chem..

[181] Fazaeli M., Yousefi S., Emam-Djomeh Z. (2013). Investigation on the effects of microwave and conventional heating methods on the phytochemicals of pomegranate (punica granatum l.) and black mulberry juices. Food Res. Int..

[182] Calani L., Beghe D., Mena P., Del Rio D., Bruni R., Fabbri A., Dall’asta C., Galaverna G. (2013). Ultra-hplc-ms(n) (poly)phenolic profiling and chemometric analysis of juices from ancient punica granatum l. Cultivars: A nontargeted approach. J. Agric. Food Chem..

[183] Turkyilmaz M., Ozkan M. (2014). Effects of condensed tannins on anthocyanins and colour of authentic pomegranate (punica granatum l.) juices. Food Chem.

[184] Sengul H., Surek E., Nilufer-Erdil D. (2014). Investigating the effects of food matrix and food components on bioaccessibility of pomegranate (punica granatum) phenolics and anthocyanins using an in-vitro gastrointestinal digestion model. Food Res. Int..

[185] Radunic M., Jukic Spika M., Goreta Ban S., Gadze J., Diaz-Perez J.C., MacLean D. (2015). Physical and chemical properties of pomegranate fruit accessions from croatia. Food Chem.

[186] Lantzouraki D.Z., Sinanoglou V.J., Tsiaka T., Proestos C., Zoumpoulakis P. (2015). Total phenolic content, antioxidant capacity and phytochemical profiling of grape and pomegranate wines. Rsc Adv..

[187] Gonzalez-Trujano M.E., Pellicer F., Mena P., Moreno D.A., Garcia-Viguera C. (2015). Antinociceptive and anti-inflammatory activities of a pomegranate (punica granatum l.) extract rich in ellagitannins. Int. J. Food Sci. Nutr..

[188] Ben-Simhon Z., Judeinstein S., Trainin T., Harel-Beja R., Bar-Ya’akov I., Borochov-Neori H., Holland D. (2015). A “white” anthocyanin-less pomegranate (punica granatum l.) caused by an insertion in the coding region of the leucoanthocyanidin dioxygenase (ldox; ans) gene. PLoS ONE.

[189] De Arajuno Santiago M.C.P., Nogueira R.I., Paim D.R.S.F., Gouvêa A.C.M.S., de Oliveira Godoy R.L., Peixoto F.M., Pacheco S., Freitas S.P. (2016). Effects of encapsulating agents on anthocyanin retention in pomegranate powder obtained by the spray drying process. LWT.

[190] Fanali C., Belluomo M.G., Cirilli M., Cristofori V., Zecchini M., Cacciola F., Russo M., Muleo R., Dugo L. (2016). Antioxidant activity evaluation and hplc-photodiode array/ms polyphenols analysis of pomegranate juice from selected italian cultivars: A comparative study. Electrophoresis.

[191] Ambigaipalan P., de Camargo A.C., Shahidi F. (2017). Identification of phenolic antioxidants and bioactives of pomegranate seeds following juice extraction using hplc-dad-esi-ms(n). Food Chem.

[192] Brighenti V., Groothuis S.F., Prencipe F.P., Amir R., Benvenuti S., Pellati F. (2017). Metabolite fingerprinting of punica granatum l. (pomegranate) polyphenols by means of high-performance liquid chromatography with diode array and electrospray ionization-mass spectrometry detection. J. Chromatogr. A.

[193] Perez-Ramirez I.F., Reynoso-Camacho R., Saura-Calixto F., Perez-Jimenez J. (2018). Comprehensive characterization of extractable and nonextractable phenolic compounds by high-performance liquid chromatography-electrospray ionization-quadrupole time-of-flight of a grape/pomegranate pomace dietary supplement. J. Agric. Food Chem..

[194] Trad M., Le Bourvellec C., Gaaliche B., Ginies C., Renard C.M.G.C., Mars M. (2013). Caprification modifies polyphenols but not cell wall concentrations in ripe figs. Sci. Hortic..

[195] Ammar S., del Mar Contreras M., Belguith-Hadrich O., Segura-Carretero A., Bouaziz M. (2015). Assessment of the distribution of phenolic compounds and contribution to the antioxidant activity in tunisian fig leaves, fruits, skins and pulps using mass spectrometry-based analysis. Food Funct..

[196] Pereira C., López-Corrales M., Serradilla M.J., del Carmen Villalobos M., Ruiz-Moyano S., Martín A. (2017). Influence of ripening stage on bioactive compounds and antioxidant activity in nine fig (ficus carica l.) varieties grown in extremadura, spain. J. Food Compos. Anal..

[197] Bochi V.C., Godoy H.T., Giusti M.M. (2015). Anthocyanin and other phenolic compounds in ceylon gooseberry (dovyalis hebecarpa) fruits. Food Chem..

[198] Carvalho A.V., Ferreira Ferreira da Silveira T., Mattietto R.A., de Padilha Oliveira M.D., Godoy H.T. (2017). Chemical composition and antioxidant capacity of acai (euterpe oleracea) genotypes and commercial pulps. J. Sci. Food Agric..

[199] Garzon G.A., Narvaez-Cuenca C.E., Vincken J.P., Gruppen H. (2017). Polyphenolic composition and antioxidant activity of acai (euterpe oleracea mart.) from colombia. Food Chem..

[200] Peris C.S., Caiado R., Lima-Filho A., Rodrigues E., Eid F., Batista Gonçalves M., de Queiroz Alves B., Guilherme Palma Urushima J., Ragazzi R., Maia M. (2018). Analysis anthocyanins extracted from the acai fruit (euterpe oleracea): A potential novel vital dye for chromovitrectomy. J. Ophthalmol..

[201] Alvarez-Suarez J.M., Giampieri F., Gasparrini M., Mazzoni L., Santos-Buelga C., Gonzalez-Paramas A.M., Forbes-Hernandez T.Y., Afrin S., Paez-Watson T., Quiles J.L. (2017). The protective effect of acerola (malpighia emarginata) against oxidative damage in human dermal fibroblasts through the improvement of antioxidant enzyme activity and mitochondrial functionality. Food Funct..

[202] Lucas-Gonzalez R., Navarro-Coves S., Pérez-Álvarez J.A., Fernández-López J., Muñoz L.A., Viuda-Martos M. (2016). Assessment of polyphenolic profile stability and changes in the antioxidant potential of maqui berry (aristotelia chilensis (molina) stuntz) during in vitro gastrointestinal digestion. Ind. Crop. Prod..

[203] Genskowsky E., Puente L.A., Perez-Alvarez J.A., Fernandez-Lopez J., Munoz L.A., Viuda-Martos M. (2016). Determination of polyphenolic profile, antioxidant activity and antibacterial properties of maqui [aristotelia chilensis (molina) stuntz] a chilean blackberry. J. Sci. Food Agric..

[204] Brauch J.E., Buchweitz M., Schweiggert R.M., Carle R. (2016). Detailed analyses of fresh and dried maqui (aristotelia chilensis (mol.) stuntz) berries and juice. Food Chem..

[205] Brauch J.E., Reuter L., Conrad J., Vogel H., Schweiggert R.M., Carle R. (2017). Characterization of anthocyanins in novel chilean maqui berry clones by hplc–dad–esi/msn and nmr-spectroscopy. J. Food Compos. Anal..

[206] Viuda-Martos M., Lucas-Gonzalez R., Ballester-Costa C., Perez-Alvarez J.A., Munoz L.A., Fernandez-Lopez J. (2018). Evaluation of protective effect of different dietary fibers on polyphenolic profile stability of maqui berry (aristotelia chilensis (molina) stuntz) during in vitro gastrointestinal digestion. Food Funct..

[207] Sommella E., Pagano F., Pepe G., Ostacolo C., Manfra M., Chieppa M., Di Sanzo R., Carabetta S., Campiglia P., Russo M. (2017). Flavonoid composition of tarocco (citrus sinensis l. Osbeck) clone “lempso” and fast antioxidant activity screening by dpph-uhplc-pda-it-tof. Phytochem Anal..

[208] Zupan A., Mikulic-Petkovsek M., Cunja V., Stampar F., Veberic R. (2013). Comparison of phenolic composition of healthy apple tissues and tissues affected by bitter pit. J. Agric. Food Chem..

[209] Wang X., Wei Z., Ma F. (2015). The effects of fruit bagging on levels of phenolic compounds and expression by anthocyanin biosynthetic and regulatory genes in red-fleshed apples. Process. Biochem..

[210] Jakobek L., Barron A.R. (2016). Ancient apple varieties from croatia as a source of bioactive polyphenolic compounds. J. Food Compos. Anal..

[211] Rehman R.N.U., You Y., Yang C., Khan A.R., Li P., Ma F. (2017). Characterization of phenolic compounds and active anthocyanin degradation in crabapple (malus orientalis) flowers. Hortic. Environ. Biotechnol..

[212] Sengul M., Eser Z., Ercıslı S. (2014). Chemical properties and antioxidant capacity of cornelian cherry genotypes grown in coruh valley of turkey. Acta Sci. Pol. Hortorum Cultus.

[213] Sozanski T., Kucharska A.Z., Szumny A., Magdalan J., Bielska K., Merwid-Lad A., Wozniak A., Dzimira S., Piorecki N., Trocha M. (2014). The protective effect of the cornus mas fruits (cornelian cherry) on hypertriglyceridemia and atherosclerosis through pparalpha activation in hypercholesterolemic rabbits. Phytomedicine.

[214] Drkenda P., Spahić A., Begić-Akagić A., Gaši F., Vranac A., Hudina M., Blanke M. (2014). Pomological characteristics of some autochthonous genotypes of cornelian cherry (cornus mas l.) in bosnia and herzegovina. Erwerbs Obstbau.

[215] Kucharska A.Z., Szumny A., Sokół-Łętowska A., Piórecki N., Klymenko S.V. (2015). Iridoids and anthocyanins in cornelian cherry (cornus mas l.) cultivars. J. Food Compos. Anal..

[216] Milenkovic Andjelkovic A., Andjelkovic M., Radovanovic A., Radovanović B., Nikolic V. (2014). Phenol composition, dpph radical scavenging antimicrobial activity cornelian cherry (cornus mas) fruit leaf extracts. Hem. Ind..

[217] Giusti M.M., Polit M.F., Ayvaz H., Tay D., Manrique I. (2014). Characterization and quantitation of anthocyanins and other phenolics in native andean potatoes. J. Agric. Food Chem..

[218] Tedesco I., Carbone V., Spagnuolo C., Minasi P., Russo G.L. (2015). Identification and quantification of flavonoids from two southern italian cultivars of allium cepa l., tropea (red onion) and montoro (copper onion), and their capacity to protect human erythrocytes from oxidative stress. J. Agric. Food Chem..

[219] Fredotović Ž., Šprung M., Soldo B., Ljubenkov I., Budić-Leto I., Bilušić T., Čikeš-Čulić V., Puizina J. (2017). Chemical composition and biological activity of allium cepa l. And allium × cornutum (clementi ex visiani 1842) methanolic extracts. Molecules.

[220] Wiczkowski W., Szawara-Nowak D., Topolska J. (2013). Red cabbage anthocyanins: Profile, isolation, identification, and antioxidant activity. Food Res. Int..

[221] Sun J., Xiao Z., Lin L.-z., Lester G.E., Wang Q., Harnly J.M., Chen P. (2013). Profiling polyphenols in five Brassica species microgreens by UHPLC-PDA-ESI/HRMS^n^. J. Agric. Food Chem..

[222] Wiczkowski W., Topolska J., Honke J. (2014). Anthocyanins profile and antioxidant capacity of red cabbages are influenced by genotype and vegetation period. J. Funct. Foods.

[223] Park S., Arasu M.V., Jiang N., Choi S.-H., Lim Y.P., Park J.-T., Al-Dhabi N.A., Kim S.-J. (2014). Metabolite profiling of phenolics, anthocyanins and flavonols in cabbage (brassica oleracea var. Capitata). Ind. Crop. Prod..

[224] Podsedek A., Redzynia M., Klewicka E., Koziolkiewicz M. (2014). Matrix effects on the stability and antioxidant activity of red cabbage anthocyanins under simulated gastrointestinal digestion. Biomed Res. Int..

[225] Wiczkowski W., Szawara-Nowak D., Topolska J. (2015). Changes in the content and composition of anthocyanins in red cabbage and its antioxidant capacity during fermentation, storage and stewing. Food Chem..

[226] Ahmadiani N., Robbins R.J., Collins T.M., Giusti M.M. (2016). Molar absorptivity (ε) and spectral characteristics of cyanidin-based anthocyanins from red cabbage. Food Chem..

[227] Socquet-Juglard D., Bennett A.A., Manns D.C., Mansfield A.K., Robbins R.J., Collins T.M., Griffiths P.D. (2016). Effects of growth temperature and postharvest cooling on anthocyanin profiles in juvenile and mature *Brassica Oleracea*. J. Agric. Food Chem..

[228] Zhang J., Wang Z., Liu X. (2017). Characterization of acylated anthocyanins in red cabbage via comprehensive two-dimensional high performance liquid chromatography and hplc-ms. J. Food Process. Preserv..

[229] Wiczkowski W., Szawara-Nowak D., Romaszko J. (2016). The impact of red cabbage fermentation on bioavailability of anthocyanins and antioxidant capacity of human plasma. Food Chem..

[230] Murador D.C., Mercadante A.Z., de Rosso V.V. (2016). Cooking techniques improve the levels of bioactive compounds and antioxidant activity in kale and red cabbage. Food Chem..

[231] Chen Y., Wang Z., Zhang H., Liu Y., Zhang S., Meng Q., Liu W. (2018). Isolation of high purity anthocyanin monomers from red cabbage with recycling preparative liquid chromatography and their photostability. Molecules.

[232] Lee H., Oh I.-N., Kim J., Jung D., Cuong N.P., Kim Y., Lee J., Kwon O., Park S.U., Lim Y. (2018). Phenolic compound profiles and their seasonal variations in new red-phenotype head-forming chinese cabbages. LWT.

[233] West M.E., Mauer L.J. (2013). Color and chemical stability of a variety of anthocyanins and ascorbic acid in solution and powder forms. J. Agric. Food Chem..

[234] Harakotr B., Suriharn B., Tangwongchai R., Scott M.P., Lertrat K. (2014). Anthocyanins and antioxidant activity in coloured waxy corn at different maturation stages. J. Funct. Foods.

[235] Huang B., Wang Z., Park J.H., Ryu O.H., Choi M.K., Lee J.Y., Kang Y.H., Lim S.S. (2015). Anti-diabetic effect of purple corn extract on c57bl/ksj db/db mice. Nutr Res. Pr..

[236] Lago C., Landoni M., Cassani E., Cantaluppi E., Doria E., Nielsen E., Giorgi A., Pilu R. (2015). Study and characterization of an ancient european flint white maize rich in anthocyanins: Millo corvo from galicia. PLoS ONE.

[237] Lao F., Giusti M.M. (2016). Quantification of purple corn (zea mays l.) anthocyanins using spectrophotometric and hplc approaches: Method comparison and correlation. Food Anal. Methods.

[238] Deineka V.I., Sidorov A.N., Deineka L.A. (2016). Determination of purple corn husk anthocyanins. J. Anal. Chem..

[239] Paucar-Menacho L.M., Martínez-Villaluenga C., Dueñas M., Frias J., Peñas E. (2017). Optimization of germination time and temperature to maximize the content of bioactive compounds and the antioxidant activity of purple corn (zea mays l.) by response surface methodology. Lwt Food Sci. Technol..

[240] Paulsmeyer M., Chatham L., Becker T., West M., West L., Juvik J. (2017). Survey of anthocyanin composition and concentration in diverse maize germplasms. J. Agric. Food Chem..

[241] Chen L., Yang M., Mou H., Kong Q. (2018). Ultrasound-assisted extraction and characterization of anthocyanins from purple corn bran. J. Food Process. Preserv..

[242] Gras C.C., Carle R., Schweiggert R.M. (2015). Determination of anthocyanins from black carrots by uhplc-pda after ultrasound-assisted extraction. J. Food Compos. Anal..

[243] Abdel-Moemin A.R. (2016). Analysis of phenolic acids and anthocyanins of pasta-like product enriched with date kernels (phoenix dactylifera l.) and purple carrots (daucus carota l. Sp. Sativus var. Atrorubens). J. Food Meas. Charact..

[244] Esatbeyoglu T., Rodriguez-Werner M., Schlosser A., Liehr M., Ipharraguerre I., Winterhalter P., Rimbach G. (2016). Fractionation of plant bioactives from black carrots (daucus carota subspecies sativus varietas atrorubens alef.) by adsorptive membrane chromatography and analysis of their potential anti-diabetic activity. J. Agric. Food Chem..

[245] Gras C.C., Bogner H., Carle R., Schweiggert R.M. (2016). Effect of genuine non-anthocyanin phenolics and chlorogenic acid on color and stability of black carrot (daucus carota ssp. Sativus var. Atrorubens alef.) anthocyanins. Food Res. Int..

[246] Carazzone C., Mascherpa D., Gazzani G., Papetti A. (2013). Identification of phenolic constituents in red chicory salads (cichorium intybus) by high-performance liquid chromatography with diode array detection and electrospray ionisation tandem mass spectrometry. Food Chem..

[247] Ferioli F., Manco M.A., D’Antuono L.F. (2015). Variation of sesquiterpene lactones and phenolics in chicory and endive germplasm. J. Food Compos. Anal..

[248] Cefola M., Carbone V., Minasi P., Pace B. (2016). Phenolic profiles and postharvest quality changes of fresh-cut radicchio ( cichorium intybus l.): Nutrient value in fresh vs. Stored leaves. J. Food Compos. Anal..

[249] Papetti A., Maietta M., Corana F., Marrubini G., Gazzani G. (2017). Polyphenolic profile of green/red spotted italian cichorium intybus salads by rp-hplc-pda-esi-msn. J. Food Compos. Anal..

[250] Tardugno R., Pozzebon M., Beggio M., Del Turco P., Pojana G. (2018). Polyphenolic profile of cichorium intybus l. Endemic varieties from the veneto region of italy. Food Chem..

[251] Slatnar A., Mikulic-Petkovsek M., Stampar F., Veberic R., Horvat J., Jakse M., Šircelj H. (2018). Game tones: Sugars, organic acids, phenolics in green purple asparagus (asparagus officinalis l.) cultivars. Turk. J. Agric. For..

[252] Jeon J., Kim J.K., Kim H., Kim Y.J., Park Y.J., Kim S.J., Kim C., Park S.U. (2018). Transcriptome analysis and metabolic profiling of green and red kale (brassica oleracea var. Acephala) seedlings. Food Chem..

[253] Papetti A., Milanese C., Zanchi C., Gazzani G. (2014). Hplc–dad–esi/msn characterization of environmentally friendly polyphenolic extract from raphanus sativus l. Var. “Cherry belle” skin and stability of its red components. Food Res. Int..

[254] Baenas N., Ferreres F., García-Viguera C., Moreno D.A. (2015). Radish sprouts—Characterization and elicitation of novel varieties rich in anthocyanins. Food Res. Int..

[255] Park C.H., Baskar T.B., Park S.Y., Kim S.J., Valan Arasu M., Al-Dhabi N.A., Kim J.K., Park S.U. (2016). Metabolic profiling and antioxidant assay of metabolites from three radish cultivars (raphanus sativus). Molecules.

[256] Mojica L., Meyer A., Berhow M.A., de Mejía E.G. (2015). Bean cultivars (phaseolus vulgaris l.) have similar high antioxidant capacity, in vitro inhibition of α-amylase and α-glucosidase while diverse phenolic composition and concentration. Food Res. Int..

[257] Jhan J.K., Chung Y.C., Chen G.H., Chang C.H., Lu Y.C., Hsu C.K. (2016). Anthocyanin contents in the seed coat of black soya bean and their anti-human tyrosinase activity and antioxidative activity. Int. J. Cosmet. Sci..

[258] Pereira-Caro G., Watanabe S., Crozier A., Fujimura T., Yokota T., Ashihara H. (2013). Phytochemical profile of a japanese black–purple rice. Food Chem..

[259] Bordiga M., Gomez-Alonso S., Locatelli M., Travaglia F., Coïsson J.D., Hermosin-Gutierrez I., Arlorio M. (2014). Phenolics characterization and antioxidant activity of six different pigmented oryza sativa l. Cultivars grown in piedmont (italy). Food Res. Int..

[260] Hao J., Zhu H., Zhang Z., Yang S., Li H. (2015). Identification of anthocyanins in black rice (oryza sativa l.) by uplc/q-tof-ms and their in vitro and in vivo antioxidant activities. J. Cereal Sci..

[261] Asem I.D., Imotomba R.K., Mazumder P.B., Laishram J.M. (2015). Anthocyanin content in the black scented rice (chakhao): Its impact on human health and plant defense. Symbiosis.

[262] Das A.B., Goud V.V., Das C. (2017). Extraction of phenolic compounds and anthocyanin from black and purple rice bran ( oryza sativa l.) using ultrasound: A comparative analysis and phytochemical profiling. Ind. Crop. Prod..

[263] Zhu Y., Sun H., He S., Lou Q., Yu M., Tang M., Tu L. (2018). Metabolism and prebiotics activity of anthocyanins from black rice (oryza sativa l.) in vitro. PLoS ONE.

[264] Zhang Y., Hu Z., Zhu M., Zhu Z., Wang Z., Tian S., Chen G. (2015). Anthocyanin accumulation and molecular analysis of correlated genes in purple kohlrabi (*Brassica oleracea* var. *gongylodes L.*). J. Agric. Food Chem..

[265] Rahim M.A., Robin A.H.K., Natarajan S., Jung H.-J., Lee J., Kim H., Kim H.-T., Park J.-I., Nou I.-S. (2018). Identification and characterization of anthocyanin biosynthesis-related genes in kohlrabi. Appl. Biochem. Biotechnol..

[266] Park C.H., Yeo H.J., Kim N.S., Eun P.Y., Kim S.-J., Arasu M.V., Al-Dhabi N.A., Park S.-Y., Kim J.K., Park S.U. (2017). Metabolic profiling of pale green and purple kohlrabi (*Brassica oleracea* var. *gongylodes L.*). Appl. Biol. Chem..

[267] Zhang Y., Hu Z., Chu G., Huang C., Tian S., Zhao Z., Chen G. (2014). Anthocyanin accumulation and molecular analysis of anthocyanin biosynthesis-associated genes in eggplant (*Solanum melongena L.*). J. Agric. Food Chem..

[268] García-Salas P., Gómez-Caravaca A.M., Morales-Soto A., Segura-Carretero A., Fernández-Gutiérrez A. (2014). Identification and quantification of phenolic compounds in diverse cultivars of eggplant grown in different seasons by high-performance liquid chromatography coupled to diode array detector and electrospray-quadrupole-time of flight-mass spectrometry. Food Res. Int..

[269] Zhang Y., Chu G., Hu Z., Gao Q., Cui B., Tian S., Wang B., Chen G. (2016). Genetically engineered anthocyanin pathway for high health-promoting pigment production in eggplant. Mol. Breed..

[270] Ferarsa S., Zhang W., Moulai-Mostefa N., Ding L., Jaffrin M.Y., Grimi N. (2018). Recovery of anthocyanins and other phenolic compounds from purple eggplant peels and pulps using ultrasonic-assisted extraction. Food Bioprod. Process..

[271] Pandino G., Meneghini M., Tavazza R., Lombardo S., Mauromicale G. (2017). Phytochemicals accumulation and antioxidant activity in callus and suspension cultures of *Cynara scolymus* L.. Plant Cell Tissue Organ Cult..

[272] Lee M.J., Park J.S., Choi D.S., Jung M.Y. (2013). Characterization and quantitation of anthocyanins in purple-fleshed sweet potatoes cultivated in korea by hplc-dad and hplc-esi-qtof-ms/ms. J. Agric. Food Chem..

[273] Xu J., Su X., Lim S., Griffin J., Carey E., Katz B., Tomich J., Smith J.S., Wang W. (2015). Characterisation and stability of anthocyanins in purple-fleshed sweet potato p40. Food Chem..

[274] Kim H.J., Park W.S., Bae J.-Y., Kang S.Y., Yang M.H., Lee S., Lee H.-S., Kwak S.-S., Ahn M.-J. (2015). Variations in the carotenoid and anthocyanin contents of korean cultural varieties and home-processed sweet potatoes. J. Food Compos. Anal..

[275] Gras C.C., Nemetz N., Carle R., Schweiggert R.M. (2017). Anthocyanins from purple sweet potato ( ipomoea batatas (l.) lam.) and their color modulation by the addition of phenolic acids and food-grade phenolic plant extracts. Food Chem..

[276] Ge J., Hu Y., Wang H., Huang Y., Zhang P., Liao Z., Chen M. (2017). Profiling of anthocyanins in transgenic purple-fleshed sweet potatoes by hplc-ms/ms: Profiling of anthocyanins in transgenic purple-fleshed sweet potatoes. J. Sci. Food Agric..

[277] Chen Y., Xu Y., Cao Y., Fang K., Xia W., Jiang Q. (2017). Combined effect of microwave and steam cooking on phytochemical compounds and antioxidant activity of purple sweet potatoes. Food Sci. Technol. Res..

[278] Wang C.J., Wang J.M., Lin W.L., Chu C.Y., Chou F.P., Tseng T.H. (2000). Protective effect of hibiscus anthocyanins against tert-butyl hydroperoxide-induced hepatic toxicity in rats. Food Chem Toxicol.

[279] Smith M.A.L., Marley K.A., Seigler D., Singletary K.W., Meline B. (2000). Bioactive properties of wild blueberry fruits. J. Food Sci..

[280] Rugină D., Sconţa Z., Leopold L., Pintea A., Bunea A., Socaciu C. (2012). Antioxidant activities of chokeberry extracts and the cytotoxic action of their anthocyanin fraction on hela human cervical tumor cells. J. Med. Food.

[281] Tsuda T., Horio F., Uchida K., Aoki H., Osawa T. (2003). Dietary cyanidin 3-o-beta-d-glucoside-rich purple corn color prevents obesity and ameliorates hyperglycemia in mice. J. Nutr..

[282] Boyer J., Brown D., Liu R.H. (2004). Uptake of quercetin and quercetin 3-glucoside from whole onion and apple peel extracts by caco-2 cell monolayers. J. Agric. Food Chem.

[283] Riboli E., Norat T. (2003). Epidemiologic evidence of the protective effect of fruit and vegetables on cancer risk. Am. J. Clin. Nutr..

[284] Kwon J.Y., Lee K.W., Hur H.J., Lee H.J. (2007). Peonidin inhibits phorbol-ester-induced cox-2 expression and transformation in jb6 p+ cells by blocking phosphorylation of erk-1 and -2. Ann. N. Y. Acad. Sci..

[285] Lazze M.C., Pizzala R., Savio M., Stivala L.A., Prosperi E., Bianchi L. (2003). Anthocyanins protect against DNA damage induced by tert-butyl-hydroperoxide in rat smooth muscle and hepatoma cells. Mutat Res..

[286] Ramirez-Tortosa C., Andersen O.M., Cabrita L., Gardner P.T., Morrice P.C., Wood S.G., Duthie S.J., Collins A.R., Duthie G.G. (2001). Anthocyanin-rich extract decreases indices of lipid peroxidation and DNA damage in vitamin e-depleted rats. Free Radic. Biol. Med..

[287] Rossi A., Serraino I., Dugo P., Di Paola R., Mondello L., Genovese T., Morabito D., Dugo G., Sautebin L., Caputi A.P. (2003). Protective effects of anthocyanins from blackberry in a rat model of acute lung inflammation. Free Radic. Res..

[288] Kong J.-M., Chia L.-S., Goh N.-K., Chia T.-F., Brouillard R. (2003). Analysis and biological activities of anthocyanins. Phytochemistry.

[289] He J., Giusti M.M. (2010). Anthocyanins: Natural colorants with health-promoting properties. Annu. Rev. Food Sci. Technol..

[290] Allen R.G., Tresini M. (2000). Oxidative stress and gene regulation. Free Radic. Biol. Med..

[291] Bors W., Heller W., Michel C., Saran M. (1990). Flavonoids as antioxidants: Determination of radical-scavenging efficiencies. Methods Enzymol..

[292] Wang H., Cao G., Prior R.L. (1997). Oxygen radical absorbing capacity of anthocyanins. J. Agric. Food Chem..

[293] Renis M., Calandra L., Scifo C., Tomasello B., Cardile V., Vanella L., Bei R., La Fauci L., Galvano F. (2008). Response of cell cycle/stress-related protein expression and DNA damage upon treatment of caco2 cells with anthocyanins. Br. J. Nutr..

[294] Parry J., Su L., Moore J., Cheng Z., Luther M., Rao J.N., Wang J.Y., Yu L.L. (2006). Chemical compositions, antioxidant capacities, and antiproliferative activities of selected fruit seed flours. J. Agric. Food Chem..

[295] Keravis T., Favot L., Abusnina A.A., Anton A., Justiniano H., Soleti R., Alabed Alibrahim E., Simard G., Andriantsitohaina R., Lugnier C. (2015). Delphinidin inhibits tumor growth by acting on vegf signalling in endothelial cells. PLoS ONE.

[296] Meyers K.J., Watkins C.B., Pritts M.P., Liu R.H. (2003). Antioxidant and antiproliferative activities of strawberries. J. Agric. Food Chem..

[297] He Y., Hu Y., Jiang X., Chen T., Ma Y., Wu S., Sun J., Jiao R., Li X., Deng L. (2017). Cyanidin-3-o-glucoside inhibits the uvb-induced ros/cox-2 pathway in hacat cells. J. Photochem. Photobiol. B Biol..

[298] Mladěnka P., Říha M., Martin J., Gorová B., Matějíček A., Spilková J. (2016). Fruit extracts of 10 varieties of elderberry (sambucus nigra l.) interact differently with iron and copper. Phytochem. Lett..

[299] Singh R.P., Dhanalakshmi S., Agarwal R. (2002). Phytochemicals as cell cycle modulators--a less toxic approach in halting human cancers. Cell Cycle.

[300] Afaq F., Adhami V.M., Mukhtar H. (2005). Photochemoprevention of ultraviolet b signaling and photocarcinogenesis. Mutat. Res..

[301] Olsson M.E., Gustavsson K.E., Andersson S., Nilsson A., Duan R.D. (2004). Inhibition of cancer cell proliferation in vitro by fruit and berry extracts and correlations with antioxidant levels. J. Agric. Food Chem..

[302] Stoner G.D., Sardo C., Apseloff G., Mullet D., Wargo W., Pound V., Singh A., Sanders J., Aziz R., Casto B. (2005). Pharmacokinetics of anthocyanins and ellagic acid in healthy volunteers fed freeze-dried black raspberries daily for 7 days. J. Clin. Pharmacol..

[303] Galvano F., La Fauci L., Lazzarino G., Fogliano V., Ritieni A., Ciappellano S., Battistini N.C., Tavazzi B., Galvano G. (2004). Cyanidins: Metabolism and biological properties. J. Nutr. Biochem..

[304] Hakimuddin F., Paliyath G., Meckling K. (2004). Selective cytotoxicity of a red grape wine flavonoid fraction against mcf-7 cells. Breast Cancer Res. Treat..

[305] Chen P.-N., Chu S.-C., Chiou H.-L., Kuo W.-H., Chiang C.-L., Hsieh Y.-S. (2006). Mulberry anthocyanins, cyanidin 3-rutinoside and cyanidin 3-glucoside, exhibited an inhibitory effect on the migration and invasion of a human lung cancer cell line. Cancer Lett..

[306] Ding M., Feng R., Wang S.Y., Bowman L., Lu Y., Qian Y., Castranova V., Jiang B.-H., Shi X. (2006). Cyanidin-3-glucoside, a natural product derived from blackberry, exhibits chemopreventive and chemotherapeutic activity. J. Biol. Chem..

[307] Roy M., Sen S., Chakraborti A.S. (2008). Action of pelargonidin on hyperglycemia and oxidative damage in diabetic rats: Implication for glycation-induced hemoglobin modification. Life Sci..

[308] Seeram N.P., Adams L.S., Zhang Y., Lee R., Sand D., Scheuller H.S., Heber D. (2006). Blackberry, black raspberry, blueberry, cranberry, red raspberry, and strawberry extracts inhibit growth and stimulate apoptosis of human cancer cells in vitro. J. Agric. Food Chem..

[309] Lim S., Xu J., Kim J., Chen T.-Y., Su X., Standard J., Carey E., Griffin J., Herndon B., Katz B. (2013). Role of anthocyanin-enriched purple-fleshed sweet potato p40 in colorectal cancer prevention. Mol. Nutr. Food Res..

[310] Shin D.Y., Lee W.S., Kim S.H., Kim M.J., Yun J.W., Lu J.N., Lee S.J., Tsoy I., Kim H.J., Ryu C.H. (2009). Anti-invasive activity of anthocyanins isolated from Vitis coignetiae in human hepatocarcinoma cells. J. Med. Food.

[311] Ali Z., Yousaf N., Larkin J. (2013). Melanoma epidemiology, biology and prognosis. Ejc Suppl..

[312] Ferlay J., Soerjomataram I., Dikshit R., Eser S., Mathers C., Rebelo M., Parkin D.M., Forman D., Bray F. (2015). Cancer incidence and mortality worldwide: Sources, methods and major patterns in globocan 2012. Int. J. Cancer.

[313] Leonardi G.C., Falzone L., Salemi R., Zanghi A., Spandidos D.A., McCubrey J.A., Candido S., Libra M. (2018). Cutaneous melanoma: From pathogenesis to therapy (review). Int. J. Oncol..

[314] Rastrelli M., Tropea S., Rossi C.R., Alaibac M. (2014). Melanoma: Epidemiology, risk factors, pathogenesis, diagnosis and classification. In vivo (Athensgreece).

[315] Ross M.I., Gershenwald J.E. (2011). Evidence-based treatment of early-stage melanoma. J. Surg. Oncol..

[316] Villanueva J., Herlyn M. (2008). Melanoma and the tumor microenvironment. Curr. Oncol. Rep..

[317] Laikova K.V., Oberemok V.V., Krasnodubets A.M., Gal’chinsky N.V., Useinov R.Z., Novikov I.A., Temirova Z.Z., Gorlov M.V., Shved N.A., Kumeiko V.V. (2019). Advances in the understanding of skin cancer: Ultraviolet radiation, mutations, and antisense oligonucleotides as anticancer drugs. Molecules.

[318] Swope V.B., Abdel-Malek Z.A. (2018). MC1R: Front and center in the bright side of dark eumelanin and DNA repair. Int. J. Mol. Sci..

[319] Serafino A., Sinibaldi-Vallebona P., Lazzarino G., Tavazzi B., Rasi G., Pierimarchi P., Andreola F., Moroni G., Galvano G., Galvano F. (2004). Differentiation of human melanoma cells induced by cyanidin-3-O-β-glucopyranoside. FASEB J..

[320] Videira I.F., Moura D.F., Magina S. (2013). Mechanisms regulating melanogenesis. An. Bras. Bermatologia.

[321] Park K.-C., Huh S.Y., Choi H.R., Kim D.-S. (2010). Biology of melanogenesis and the search for hypopigmenting agents. Dermatol. Sin..

[322] Kanitakis J. (2002). Anatomy, histology and immunohistochemistry of normal human skin. Eur. J. Dermatol. Ejd.

[323] Farage M.A., Miller K.W., Berardesca E., Maibach H.I. (2009). Clinical implications of aging skin: Cutaneous disorders in the elderly. Am. J. Clin. Dermatol..

[324] Pennello G., Devesa S., Gail M. (2000). Association of surface ultraviolet b radiation levels with melanoma and nonmelanoma skin cancer in United States blacks. Cancer Epidemiol. Biomark. Prev..

[325] Leonel E., Rojo D.E.R., Graf B., Diana, Chung M., Ribnicky D., Fridlender B., Raskin I., Wallace T.C., Giusti M.M. (2013). Role of Anthocyanins in Skin Aging and UV Induced Skin Damage. Anthocyanins in Health and Disease.

[326] Bauer J., Garbe C. (2003). Acquired melanocytic nevi as risk factor for melanoma development. A comprehensive review of epidemiological data. Pigment Cell Res..

[327] Hawkes J.E., Truong A., Meyer L.J. (2016). Genetic predisposition to melanoma. Semin. Oncol..

[328] Russo A.E., Torrisi E., Bevelacqua Y., Perrotta R., Libra M., McCubrey J.A., Spandidos D.A., Stivala F., Malaponte G. (2009). Melanoma: Molecular pathogenesis and emerging target therapies (review). Int. J. Oncol..

[329] Hirst N.G., Gordon L.G., Scuffham P.A., Green A.C. (2012). Lifetime cost-effectiveness of skin cancer prevention through promotion of daily sunscreen use. Value Health.

[330] Ives N.J., Stowe R.L., Lorigan P., Wheatley K. (2007). Chemotherapy compared with biochemotherapy for the treatment of metastatic melanoma: A meta-analysis of 18 trials involving 2621 patients. J. Clin. Oncol..

[331] Bishayee A. (2009). Cancer prevention and treatment with resveratrol: From rodent studies to clinical trials. Cancer Prev. Res..

[332] Aquilato A., Lopez V., Doonan B., Hsieh T.-C., Pinto J.T., Wu E., Wu J.M. (2014). Chapter 102—Braf Mutation in Melanoma and Dietary Polyphenols as Adjunctive Treatment Strategy. Polyphenols in Human Health Disease.

[333] Shishodia S., Aggarwal B.B., Aggarwal B.B., Shishodia S. (2006). Resveratrol: A polyphenol for all seasons. Resveratrol in Health Disease.

[334] Imtiaz A., Siddiqui R.S.T., Chamcheu J.C., Mukhtar H., Xi Y. (2011). Bioactive Food Components For Melanoma: An Overview, Skin Cancer Overview.

[335] Chinembiri T.N., du Plessis L.H., Gerber M., Hamman J.H., du Plessis J. (2014). Review of natural compounds for potential skin cancer treatment. Molecules.

[336] Giampieri F., Alvarez-Suarez J.M., Tulipani S., Gonzàles-Paramàs A.M., Santos-Buelga C., Bompadre S., Quiles J.L., Mezzetti B., Battino M. (2012). Photoprotective potential of strawberry (fragaria × ananassa) extract against uv-a irradiation damage on human fibroblasts. J. Agric. Food Chem..

[337] Saewan N., Jimtaisong A. (2013). Photoprotection of natural flavonoids. J. Appl. Pharm. Sci..

[338] Syed D.N., Afaq F., Maddodi N., Johnson J.J., Sarfaraz S., Ahmad A., Setaluri V., Mukhtar H. (2011). Inhibition of human melanoma cell growth by the dietary flavonoid fisetin is associated with disruption of wnt/[beta]-catenin signaling and decreased mitf levels. J. Invest. Dermatol..

[339] Murapa P., Dai J., Chung M., Mumper R.J., D’Orazio J. (2012). Anthocyanin-rich fractions of blackberry extracts reduce uv-induced free radicals and oxidative damage in keratinocytes. Phytother. Res..

[340] Bae J.Y., Lim S.S., Kim S.J., Choi J.S., Park J., Ju S.M., Han S.J., Kang I.J., Kang Y.H. (2009). Bog blueberry anthocyanins alleviate photoaging in ultraviolet-b irradiation-induced human dermal fibroblasts. Mol. Nutr. Food Res..

[341] Afaq F., Katiyar S.K. (2011). Polyphenols: Skin photoprotection and inhibition of photocarcinogenesis. Mini Rev. Med. Chem..

[342] Harborne J.B. (1958). Spectral methods of characterizing anthocyanins. Biochem. J..

[343] Chan C.F., Lien C.Y., Lai Y.C., Huang C.L., Liao W.C. (2010). Influence of purple sweet potato extracts on the uv absorption properties of a cosmetic cream. J. Cosmet. Sci..

[344] Tsoyi K., Park H.B., Kim Y.M., Chung J.I., Shin S.C., Shim H.J., Lee W.S., Seo H.G., Lee J.H., Chang K.C. (2008). Protective effect of anthocyanins from black soybean seed coats on uvb-induced apoptotic cell death in vitro and in vivo. J. Agric. Food Chem..

[345] Miko Enomoto T., Johnson T., Peterson N., Homer L., Walts D., Johnson N. (2005). Combination glutathione and anthocyanins as an alternative for skin care during external-beam radiation. Am. J. Surg..

[346] Gómez-Cordovés C., Bartolomé B., Vieira W., Virador V.M. (2001). Effects of wine phenolics and sorghum tannins on tyrosinase activity and growth of melanoma cells. J. Agric. Food Chem..

[347] Lu H., Li J., Zhang D., Stoner G.D., Huang C. (2006). Molecular mechanisms involved in chemoprevention of black raspberry extracts: From transcription factors to their target genes. Nutr. Cancer.

[348] Choung M.G., Hwang Y.S., Kim G.P., Ahn K.G., Shim H.S., Hong S.B., Choi J.H., Yu C.Y., Chung I.M., Kim S.H. (2013). Antimelanogenic effect and whitening of anthocyanin rich fraction from seeds of liriope platyphylla. Korean J. Med. Crop. Sci..

[349] Hwang J.-M., Kuo H.-C., Lin C.-T., Kao E.-S. (2013). Inhibitory effect of liposome-encapsulated anthocyanin on melanogenesis in human melanocytes. Pharm. Biol..

[350] Razina T.G., Zueva E.P., Ulrich A.V., Rybalkina O.Y., Chaikovskii A.V., Isaikina N.V., Kalinkina G.I., Zhdanov V.V., Zyuz’kov G.N. (2016). Antitumor effects of sorbus aucuparia l. Extract highly saturated with anthocyans and their mechanisms. Bull. Exp. Biol. Med..

[351] George B.P.A., Abrahamse H., Hemmaragala N.M. (2016). Caspase dependent apoptotic inhibition of melanoma and lung cancer cells by tropical rubus extracts. Biomed. Pharmacother..

[352] Yanarojana M., Nararatwanchai T., Thairat S., Tancharoen S. (2017). Antiproliferative activity and induction of apoptosis in human melanoma cells by houttuynia cordata thunb extract. Anticancer Res..

[353] Kanlayavattanakul M., Lourith N., Chaikul P. (2018). Biological activity and phytochemical profiles of dendrobium: A new source for specialty cosmetic materials. Ind. Crop. Prod..

[354] Su C.-C., Wang C.-J., Huang K.-H., Lee Y.-J., Chan W.-M., Chang Y.-C. (2018). Anthocyanins from hibiscus sabdariffa calyx attenuate in vitro and in vivo melanoma cancer metastasis. J. Funct. Foods.

[355] Forni C., Frattarelli A., Lentini A., Beninati S., Lucioli S., Caboni E. (2016). Assessment of the antiproliferative activity on murine melanoma cells of extracts from elicited cell suspensions of strawberry, strawberry tree, blackberry and red raspberry. Plant Biosyst..

[356] Afaq F., Syed D.N., Malik A., Hadi N., Sarfaraz S., Kweon M.-H., Khan N., Zaid M.A., Mukhtar H. (2007). Delphinidin, an anthocyanidin in pigmented fruits and vegetables, protects human hacat keratinocytes and mouse skin against uvb-mediated oxidative stress and apoptosis. J. Investig. Dermatol..

[357] Zillich O.V., Schweiggert-Weisz U., Eisner P., Kerscher M. (2015). Polyphenols as active ingredients for cosmetic products. Int. J. Cosmet. Sci..

[358] Draelos Z.D. (2014). Hair, sun, regulation, and beauty. J. Cosmet. Dermatol..

[359] Pillai S., Cornell M., Oresajo C. (2018). Epidermal Barrier Part 1: Skin Physiology Pertinent to Cosmetic Dermatology.

[360] Darlenski R., Kazandjieva J., Hristakieva E., Fluhr J.W. (2014). Atopic dermatitis as a systemic disease. Clin. Dermatol..

[361] Brown S.J., McLean W.H. (2012). One remarkable molecule: Filaggrin. J. Investig. Dermatol..

[362] Moser K., Kriwet K., Naik A., Kalia Y.N., Guy R.H. (2001). Passive skin penetration enhancement and its quantification in vitro. Eur. J. Pharm. Biopharm..

[363] Elias P.M. (2008). Skin barrier function. Curr. Allergy Asthma Rep..

[364] Trommer H., Neubert R.H.H. (2006). Overcoming the stratum corneum: The modulation of skin penetration. Ski. Pharmacol. Physiol..

[365] Westfall A. (2015). Evaluation of the Efficacy of Anthocyanins as Biologically Active Ingredients in Lipstick Formulations. Master’s Thesis.

[366] Lai J., Xin C., Zhao Y., Feng B., He C., Dong Y., Fang Y., Wei S. (2012). Study of active ingredients in black soybean sprouts and their safety in cosmetic use. Molecules.

[367] Plundrich N., Grace M.H., Raskin I., Ann Lila M. (2013). Bioactive polyphenols from muscadine grape and blackcurrant stably concentrated onto protein-rich matrices for topical applications. Int. J. Cosmet. Sci..

